# Supplementary material for: Surgical procedures in Danish children 1999–2018
Source: PLoS One. 2023 Apr 27;18(4):e0285047. doi: 10.1371/journal.pone.0285047 (PMC10138199; doi:10.1371/journal.pone.0285047)
Supplement: S1 File — (DOCX) [file pone.0285047.s001.docx]

**Table A in S1 File. All inpatient surgical procedures in Danish children, 0-5 years of age, 1999-2018.**

|  | Events  Rate (per 1000)  IRR (95% CI) vs. 1999 | **Age** | | | **Sex** | |
| --- | --- | --- | --- | --- | --- | --- |
| **Year** | **Total** | **0-28 days** | **28-365 days** | **1-5 years** | **Female** | **Male** |
| 1999 | 8833  25.98  Ref. year for IRR | 430  84.31 | 1829  29.71 | 6574  24.05 | 3160  19.12 | 5673  32.47 |
| 2000 | 9497  28.22  1.09 (1.06-1.12) | 579  112.40  1.33 (1.18-1.51) | 2090  34.00  1.14 (1.07-1.22) | 6828  25.30  1.05 (1.02-1.09) | 3484  21.30  1.11 (1.06-1.17) | 6013  34.76  1.07 (1.03-1.11) |
| 2001 | 9657  28.83  1.11 (1.08-1.14) | 594  117.46  1.39 (1.23-1.58) | 2264  36.67  1.23 (1.16-1.31) | 6799  25.35  1.05 (1.02-1.09) | 3617  22.18  1.16 (1.11-1.22) | 6040  35.13  1.08 (1.04-1.12) |
| 2002 | 9389  28.32  1.09 (1.06-1.12) | 621  125.76  1.49 (1.32-1.69) | 1929  32.16  1.08 (1.02-1.15) | 6839  25.65  1.07 (1.03-1.10) | 3507  21.71  1.14 (1.08-1.19) | 5882  34.59  1.07 (1.03-1.11) |
| 2003 | 9292  28.23  1.09 (1.06-1.12) | 630  126.40  1.50 (1.33-1.69) | 2042  34.38  1.16 (1.09-1.23) | 6620  25.00  1.04 (1.00-1.08) | 3475  21.68  1.13 (1.08-1.19) | 5817  34.46  1.06 (1.02-1.10) |
| 2004 | 9695  29.61  1.14 (1.11-1.17) | 740  148.92  1.77 (1.57-1.99) | 2159  35.94  1.21 (1.14-1.29) | 6796  25.90  1.08 (1.04-1.11) | 3817  23.93  1.25 (1.19-1.31) | 5878  35.00  1.08 (1.04-1.12) |
| 2005 | 9573  29.38  1.13 (1.10-1.16) | 743  149.38  1.77 (1.57-2.00) | 2216  36.96  1.24 (1.17-1.32) | 6614  25.35  1.05 (1.02-1.09) | 3816  24.04  1.26 (1.20-1.32) | 5757  34.46  1.06 (1.02-1.10) |
| 2006 | 9384  28.97  1.12 (1.08-1.15) | 628  125.04  1.48 (1.31-1.68) | 2129  35.53  1.20 (1.12-1.27) | 6627  25.59  1.06 (1.03-1.10) | 3612  22.87  1.20 (1.14-1.25) | 5772  34.77  1.07 (1.03-1.11) |
| 2007 | 9362  28.90  1.11 (1.08-1.15) | 739  148.93  1.77 (1.57-1.99) | 2225  37.12  1.25 (1.17-1.33) | 6398  24.70  1.03 (0.99-1.06) | 3608  22.86  1.20 (1.14-1.25) | 5754  34.65  1.07 (1.03-1.11) |
| 2008 | 7975  24.55  0.95 (0.92-0.97) | 682  135.69  1.61 (1.43-1.82) | 1787  29.64  1.00 (0.93-1.06) | 5506  21.21  0.88 (0.85-0.91) | 3025  19.10  1.00 (0.95-1.05) | 4950  29.74  0.92 (0.88-0.95) |
| 2009 | 8970  27.65  1.06 (1.03-1.10) | 639  131.63  1.56 (1.38-1.76) | 1939  32.47  1.09 (1.03-1.16) | 6392  24.60  1.02 (0.99-1.06) | 3432  21.72  1.14 (1.08-1.19) | 5538  33.28  1.02 (0.99-1.06) |
| 2010 | 9287  28.73  1.11 (1.07-1.14) | 730  148.44  1.76 (1.56-1.98) | 1934  32.85  1.11 (1.04-1.18) | 6623  25.52  1.06 (1.03-1.10) | 3412  21.68  1.13 (1.08-1.19) | 5875  35.41  1.09 (1.05-1.13) |
| 2011 | 9005  28.12  1.08 (1.05-1.11) | 752  163.97  1.94 (1.73-2.19) | 1813  31.62  1.06 (1.00-1.14) | 6440  24.93  1.04 (1.00-1.07) | 3352  21.48  1.12 (1.07-1.18) | 5653  34.42  1.06 (1.02-1.10) |
| 2012 | 8842  28.15  1.08 (1.05-1.12) | 830  185.31  2.20 (1.96-2.47) | 1804  33.25  1.12 (1.05-1.19) | 6208  24.31  1.01 (0.98-1.05) | 3375  22.04  1.15 (1.10-1.21) | 5467  33.96  1.05 (1.01-1.09) |
| 2013 | 9008  29.40  1.13 (1.10-1.17) | 820  189.51  2.25 (2.00-2.53) | 1967  36.90  1.24 (1.17-1.32) | 6221  25.01  1.04 (1.00-1.08) | 3437  23.01  1.20 (1.15-1.26) | 5571  35.47  1.09 (1.05-1.13) |
| 2014 | 8460  28.36  1.09 (1.06-1.12) | 876  199.37  2.36 (2.11-2.65) | 1877  36.00  1.21 (1.14-1.29) | 5707  23.60  0.98 (0.95-1.02) | 3170  21.78  1.14 (1.08-1.20) | 5290  34.62  1.07 (1.03-1.11) |
| 2015 | 7879  26.99  1.04 (1.01-1.07) | 879  197.17  2.34 (2.08-2.62) | 1890  35.75  1.20 (1.13-1.28) | 5110  21.78  0.91 (0.87-0.94) | 2960  20.79  1.09 (1.03-1.14) | 4919  32.89  1.01 (0.98-1.05) |
| 2016 | 7594  26.18  1.01 (0.98-1.04) | 1067  226.45  2.69 (2.40-3.00) | 1774  32.04  1.08 (1.01-1.15) | 4753  20.66  0.86 (0.83-0.89) | 2884  20.43  1.07 (1.02-1.012) | 4710  31.63  0.97 (0.94-1.01) |
| 2017 | 7586  25.87  1.00 (0.97-1.03) | 1143  239.89  2.85 (2.55-3.18) | 1901  33.27  1.12 (1.05-1.19) | 4542  19.63  0.82 (0.79-0.85) | 2957  20.74  1.09 (1.03-1.14) | 4629  30.73  0.95 (0.91-0.98) |
| 2018 | 7727  25.96  1.00 (0.97-1.03) | 1140  239.40  2.84 (2.54-3.17) | 1782  30.88  1.04 (0.97-1.11) | 4805  20.44  0.85 (0.82-0.88) | 3011  20.82  1.09 (1.04-1.14) | 4716  30.89  0.95 (0.92-0.99) |
| Total | 177,015  27.84 | 15,262  158.26 | 39,351  33.84 | 122,402  24.01 | 67,111  21.67 | 109,904  33.70 |

Each cell contains: (1) the number of events, (2) the incidence rate (per 1000 person-years), and (3) the incidence rate ratios (IRR) with 95% confidence intervals (CI) compared to the reference year 1999. Left-hand side of table: The totals of (1)-(3) are presented by calendar year. Right-hand side of table: For each calendar year (1)-(3) are presented by age group and sex.

**Table B in S1 File. Inpatient surgical procedures by specialties (lower) in 0–5-year-old Danish children, 1999-2018.**

|  | Events  Rate (per 1000)  IRR (95% CI) vs. 1999 | **Surgical specialties** | | | | |
| --- | --- | --- | --- | --- | --- | --- |
| **Year** | **All surgeries** | **Paediatric** | **Orthopaedic** | **Cardiothoracic** | **Vascular** | **Gynaecologic** |
| 1999 | 8833  25.98  Ref. year for IRR | 2198  6.46 | 1140  3.35 | 512  1.51 | 62  0.18 | 24  0.07 |
| 2000 | 9497  28.22  1.09 (1.06-1.12) | 2420  7.19  1.11 (1.05-1.18) | 1261  3.75  1.12 (1.03-1.21) | 551  1.64  1.09 (0.96-1.23) | 52  0.15  0.85 (0.59-1.22) | 19  0.06  0.80 (0.44-1.46) |
| 2001 | 9657  28.83  1.11 (1.08-1.14) | 2216  6.61  1.02 (0.96-1.09) | 1128  3.36  1.00 (0.92-1.09) | 571  1.70  1.13 (1.00-1.27) | 56  0.17  0.92 (0.64-1.31) | 28  0.08  1.18 (0.69-2.04) |
| 2002 | 9389  28.32  1.09 (1.06-1.12) | 2084  6.28  0.97 (0.92-1.03) | 1145  3.45  1.03 (0.95-1.12) | 564  1.70  1.13 (1.00-1.27) | 55  0.17  0.91 (0.63-1.31) | 35  0.11  1.49 (0.89-2.51) |
| 2003 | 9292  28.23  1.09 (1.06-1.12) | 2106  6.39  0.99 (0.93-1.05) | 1076  3.26  0.97 (0.90-1.06) | 585  1.78  1.18 (1.05-1.33) | 67  0.20  1.11 (0.79-1.57) | 27  0.08  1.16 (0.67-2.01) |
| 2004 | 9695  29.61  1.14 (1.11-1.17) | 2061  6.29  0.97 (0.92-1.03) | 1053  3.21  0.96 (0.88-1.04) | 629  1.92  1.27 (1.13-1.43) | 64  0.20  1.07 (0.75-1.52) | 27  0.08  1.17 (0.67-2.02) |
| 2005 | 9573  29.38  1.13 (1.10-1.16) | 2005  6.15  0.95 (0.90-1.01) | 1075  3.29  0.98 (0.90-1.07) | 662  2.03  1.35 (1.20-1.51) | 67  0.21  1.13 (0.80-1.59) | 46  0.14  2.00 (1.22-3.27) |
| 2006 | 9384  28.97  1.12 (1.08-1.15) | 2064  6.36  0.98 (0.93-1.05) | 1090  3.36  1.00 (0.92-1.09) | 650  2.00  1.33 (1.19-1.49) | 50  0.15  0.85 (0.58-1.23) | 46  0.14  2.01 (1.23-3.29) |
| 2007 | 9362  28.90  1.11 (1.08-1.15) | 2182  6.73  1.04 (0.98-1.10) | 1105  3.41  1.02 (0.94-1.10) | 652  2.01  1.34 (1.19-1.50) | 44  0.14  0.74 (0.51-1.09) | 56  0.17  2.45 (1.52-3.95) |
| 2008 | 7975  24.55  0.95 (0.92-0.97) | 1931  5.94  0.92 (0.86-0.98) | 1013  3.11  0.93 (0.85-1.01) | 570  1.75  1.16 (1.03-1.31) | 46  0.14  0.78 (0.53-1.14) | 35  0.11  1.52 (0.91-2.56) |
| 2009 | 8970  27.65  1.06 (1.03-1.10) | 2228  6.86  1.06 (1.00-1.13) | 1033  3.18  0.95 (0.87-1.03) | 563  1.73  1.15 (1.02-1.30) | 49  0.15  0.83 (0.57-1.20) | 41  0.13  1.79 (1.08-2.96) |
| 2010 | 9287  28.73  1.11 (1.07-1.14) | 2350  7.26  1.12 (1.06-1.19) | 1037  3.20  0.96 (0.88-1.04) | 597  1.84  1.23 (1.09-1.38) | 50  0.15  0.85 (0.58-1.23) | 31  0.10  1.36 (0.80-2.31) |
| 2011 | 9005  28.12  1.08 (1.05-1.11) | 2363  7.37  1.14 (1.08-1.21) | 1207  3.76  1.12 (1.04-1.22) | 582  1.82  1.21 (1.07-1.36) | 63  0.20  1.08 (0.76-1.53) | 23  0.07  1.02 (0.57-1.80) |
| 2012 | 8842  28.15  1.08 (1.05-1.12) | 2241  7.13  1.10 (1.04-1.17) | 1122  3.57  1.06 (0.98-1.16) | 626  1.99  1.32 (1.18-1.49) | 48  0.15  0.84 (0.57-1.22) | 32  0.10  1.44 (0.85-2.45) |
| 2013 | 9008  29.40  1.13 (1.10-1.17) | 2462  8.03  1.24 (1.17-1.32) | 1182  3.85  1.15 (1.06-1.25) | 594  1.94  1.29 (1.14-1.45) | 56  0.18  1.00 (0.70-1.44) | 24  0.08  1.11 (0.63-1.95) |
| 2014 | 8460  28.36  1.09 (1.06-1.12) | 2284  7.65  1.18 (1.12-1.25) | 1049  3.51  1.05 (0.96-1.14) | 608  2.04  1.35 (1.20-1.52) | 43  0.14  0.79 (0.53-1.16) | 17  0.06  0.81 (0.43-1.50) |
| 2015 | 7879  26.99  1.04 (1.01-1.07) | 2212  7.57  1.17 (1.10-1.24) | 973  3.33  0.99 (0.91-1.08) | 565  1.93  1.28 (1.14-1.45) | 72  0.25  1.35 (0.96-1.90) | 25  0.09  1.21 (0.69-2.12) |
| 2016 | 7594  26.18  1.01 (0.98-1.04) | 2033  7.00  1.08 (1.02-1.15) | 980  3.37  1.01 (0.92-1.10) | 566  1.95  1.29 (1.15-1.46) | 57  0.20  1.08 (0.75-1.54) | 26  0.09  1.27 (0.73-2.21) |
| 2017 | 7586  25.87  1.00 (0.97-1.03) | 2012  6.86  1.06 (1.00-1.13) | 942  3.21  0.96 (0.88-1.04) | 671  2.29  1.52 (1.35-1.70) | 44  0.15  0.82 (0.56-1.21) | 13  0.04  0.63 (0.32-1.23) |
| 2018 | 7727  25.96  1.00 (0.97-1.03) | 1788  6.00  0.93 (0.87-0.99) | 909  3.05  0.91 (0.83-0.99) | 661  2.22  1.47 (1.31-1.65) | 26  0.09  0.48 (0.30-0.76) | 30  0.10  1.43 (0.83-2.44) |
| Total | 177,015  27.84 | 43,240  6.73 | 21,520  3.38 | 11,979  1.88 | 1071  0.17 | 605  0.10 |

Each cell contains: (1) the number of events, (2) the incidence rate (per 1000 person-years), and (3) the incidence rate ratios (IRR) with 95% Confidence intervals (CI) compared to the reference year 1999. Left-hand side of table: The totals of (1)-(3) are presented by calendar year. Right-hand side of table: For each calendar year (1)-(3) are presented by five different surgical specialties ().

**Table C in S1 File. Inpatient surgical procedures by specialties (upper) in Danish children, 0-5 years of age, 1999-2018.**

|  | Events  Rate (per 1000)  IRR (95% CI) vs. 1999 | **Surgical specialties** | | | | |
| --- | --- | --- | --- | --- | --- | --- |
| **Year** | **All surgeries** | **Otorhinolaryngology** | **Plastic** | **Neurological** | **Eye** | **Oral** |
| 1999 | 8833  25.98  Ref. year for IRR | 3078  9.05 | 595  1.75 | 269  0.79 | 362  1.06 | 97  0.29 |
| 2000 | 9497  28.22  1.09 (1.06-1.12) | 3176  9.43  1.04 (0.99-1.10) | 774  2.30  1.31 (1.18-1.46) | 284  0.84  1.07 (0.90-1.26) | 319  0.95  0.89 (0.77-1.03) | 103  0.31  1.07 (0.81-1.42) |
| 2001 | 9657  28.83  1.11 (1.08-1.14) | 3342  9.97  1.10 (1.05-1.16) | 755  2.25  1.29 (1.16-1.43) | 287  0.86  1.08 (0.92-1.28) | 342  1.02  0.96 (0.83-1.11) | 95  0.28  0.99 (0.75-1.32) |
| 2002 | 9389  28.32  1.09 (1.06-1.12) | 3359  10.12  1.12 (1.06-1.17) | 692  2.08  1.19 (1.07-1.33) | 262  0.79  1.00 (0.84-1.18) | 291  0.88  0.82 (0.71-0.96) | 80  0.24  0.84 (0.63-1.14) |
| 2003 | 9292  28.23  1.09 (1.06-1.12) | 3267  9.91  1.10 (1.04-1.15) | 605  1.84  1.05 (0.94-1.17) | 259  0.79  0.99 (0.84-1.18) | 290  0.88  0.83 (0.71-0.96) | 67  0.20  0.71 (0.52-0.97) |
| 2004 | 9695  29.61  1.14 (1.11-1.17) | 3559  10.85  1.20 (1.14-1.26) | 583  1.78  1.02 (0.91-1.14) | 244  0.74  0.94 (0.79-1.12) | 282  0.86  0.81 (0.69-0.94) | 56  0.17  0.60 (0.43-0.83) |
| 2005 | 9573  29.38  1.13 (1.10-1.16) | 3706  11.36  1.26 (1.20-1.32) | 593  1.82  1.04 (0.93-1.16) | 239  0.73  0.93 (0.78-1.10) | 270  0.83  0.78 (0.66-0.91) | 45  0.14  0.48 (0.34-0.69) |
| 2006 | 9384  28.97  1.12 (1.08-1.15) | 3666  11.30  1.25 (1.19-1.31) | 627  1.93  1.10 (0.99-1.24) | 234  0.72  0.91 (0.77-1.09) | 251  0.77  0.73 (0.62-0.85) | 40  0.12  0.43 (0.30-0.62) |
| 2007 | 9362  28.90  1.11 (1.08-1.15) | 3585  11.05  1.22 (1.16-1.28) | 596  1.84  1.05 (0.94-1.18) | 256  0.79  1.00 (0.84-1.18) | 253  0.78  0.73 (0.62-0.86) | 25  0.08  0.27 (0.17-0.42) |
| 2008 | 7975  24.55  0.95 (0.92-0.97) | 2709  8.33  0.92 (0.87-0.97) | 573  1.76  1.01 (0.90-1.13) | 245  0.75  0.95 (0.80-1.13) | 215  0.66  0.62 (0.52-0.73) | 33  0.10  0.36 (0.24-0.53) |
| 2009 | 8970  27.65  1.06 (1.03-1.10) | 3267  10.06  1.11 (1.06-1.17) | 576  1.77  1.01 (0.90-1.14) | 232  0.71  0.90 (0.76-1.08) | 225  0.69  0.65 (0.55-0.77) | 41  0.13  0.44 (0.31-0.64) |
| 2010 | 9287  28.73  1.11 (1.07-1.14) | 3340  10.32  1.14 (1.09-1.20) | 605  1.87  1.07 (0.95-1.20) | 220  0.68  0.86 (0.72-1.03) | 220  0.68  0.64 (0.54-0.75) | 35  0.11  0.38 (0.26-0.56) |
| 2011 | 9005  28.12  1.08 (1.05-1.11) | 2974  9.28  1.02 (0.97-1.08) | 555  1.73  0.99 (0.88-1.11) | 224  0.70  0.88 (0.74-1.05) | 195  0.61  0.57 (0.48-0.68) | 39  0.12  0.43 (0.29-0.62) |
| 2012 | 8842  28.15  1.08 (1.05-1.12) | 3001  9.54  1.05 (1.00-1.11) | 588  1.87  1.07 (0.95-1.20) | 233  0.74  0.94 (0.79-1.12) | 166  0.53  0.50 (0.41-0.60) | 32  0.10  0.36 (0.24-0.53) |
| 2013 | 9008  29.40  1.13 (1.10-1.17) | 2952  9.62  1.06 (1.01-1.12) | 587  1.91  1.09 (0.98-1.23) | 238  0.78  0.98 (0.82-1.17) | 160  0.52  0.49 (0.41-0.59) | 33  0.11  0.38 (0.25-0.56) |
| 2014 | 8460  28.36  1.09 (1.06-1.12) | 2822  9.45  1.04 (0.99-1.10) | 526  1.76  1.01 (0.89-1.13) | 213  0.71  0.90 (0.75-1.08) | 205  0.69  0.64 (0.54-0.76) | 32  0.11  0.38 (0.25-0.56) |
| 2015 | 7879  26.99  1.04 (1.01-1.07) | 2656  9.09  1.00 (0.95-1.06) | 472  1.61  0.92 (0.82-1.04) | 188  0.64  0.81 (0.67-0.98) | 174  0.60  0.56 (0.47-0.67) | 18  0.06  0.22 (0.13-0.36) |
| 2016 | 7594  26.18  1.01 (0.98-1.04) | 2555  8.80  0.97 (0.92-1.02) | 464  1.60  0.91 (0.81-1.03) | 180  0.62  0.78 (0.65-0.95) | 159  0.55  0.51 (0.43-0.62) | 18  0.06  0.22 (0.13-0.36) |
| 2017 | 7586  25.87  1.00 (0.97-1.03) | 2490  8.48  0.94 (0.89-0.99) | 382  1.30  0.74 (0.65-0.85) | 214  0.73  0.92 (0.77-1.10) | 155  0.53  0.50 (0.41-0.60) | 26  0.09  0.31 (0.20-0.48) |
| 2018 | 7727  25.96  1.00 (0.97-1.03) | 2687  9.02  1.00 (0.95-1.05) | 500  1.68  0.96 (0.85-1.08) | 237  0.80  1.01 (0.84-1.20) | 160  0.54  0.50 (0.42-0.61) | 28  0.09  0.33 (0.22-0.50) |
| Total | 177,015  27.84 | 62,191  9.77 | 11,648  1.83 | 4758  0.75 | 4694  0.74 | 943  0.14 |

Each cell contains: (1) the number of events, (2) the incidence rate (per 1000 person-years), and (3) the incidence rate ratios (IRR) with 95% Confidence intervals (CI) compared to the reference year 1999. Left-hand side of table: The totals of (1)-(3) are presented by calendar year. Right-hand side of table: For each calendar year (1)-(3) are presented by five different surgical specialties.

**Table D in S1 File. Minor inpatient surgical procedures in Danish children, 0-5 years of age, 1999-2018.**

|  | Events  Rate (per 1000)  IRR (95% CI) vs. 1999 | **Age** | | | **Sex** | |
| --- | --- | --- | --- | --- | --- | --- |
| **Year** | **Total** | **0-28 days** | **28-365 days** | **1-5 years** | **Female** | **Male** |
| 1999 | 947  2.78  Ref. year for IRR | 115  22.55 | 231  3.75 | 601  2.20 | 375  2.27 | 572  3.27 |
| 2000 | 1031  3.06  1.10 (1.01-1.20) | 133  25.82  1.15 (0.89-1.47) | 244  3.97  1.06 (0.88-1.27) | 654  2.42  1.10 (0.99-1.23) | 418  2.55  1.13 (0.98-1.29) | 613  3.54  1.08 (0.97-1.21) |
| 2001 | 1307  3.90  1.40 (1.29-1.52) | 201  39.74  1.76 (1.40-2.22) | 300  4.86  1.29 (1.09-1.54) | 806  3.00  1.37 (1.23-1.52) | 481  2.95  1.30 (1.14-1.49) | 826  4.80  1.47 (1.32-1.63) |
| 2002 | 1237  3.73  1.34 (1.23-1.46) | 180  36.44  1.62 (1.28-2.04) | 309  5.15  1.37 (1.16-1.63) | 748  2.80  1.28 (1.15-1.42) | 487  3.01  1.33 (1.16-1.52) | 750  4.41  1.35 (1.21-1.50) |
| 2003 | 1331  4.04  1.45 (1.33-1.58) | 199  39.92  1.77 (1.41-2.23) | 335  5.64  1.50 (1.27-1.78) | 797  3.01  1.37 (1.23-1.52) | 525  3.27  1.44 (1.26-1.65) | 806  4.77  1.46 (1.31-1.62) |
| 2004 | 1564  4.77  1.71 (1.58-1.86) | 229  46.08  2.04 (1.63-2.56) | 379  6.30  1.68 (1.43-1.98) | 956  3.64  1.66 (1.49-1.83) | 706  4.42  1.95 (1.72-2.21) | 858  5.10  1.56 (1.40-1.73) |
| 2005 | 1331  4.08  1.47 (1.35-1.59) | 151  30.35  1.35 (1.06-1.72) | 360  6.00  1.60 (1.36-1.89) | 820  3.14  1.43 (1.29-1.59) | 605  3.81  1.68 (1.48-1.91) | 726  4.34  1.33 (1.19-1.48) |
| 2006 | 1166  3.60  1.29 (1.19-1.41) | 93  18.51  0.82 (0.62-1.08) | 297  4.95  1.32 (1.11-1.57) | 776  2.99  1.36 (1.22-1.51) | 482  3.05  1.34 (1.17-1.54) | 684  4.12  1.26 (1.13-1.41) |
| 2007 | 1017  3.14  1.13 (1.03-1.23) | 110  22.16  0.98 (0.76-1.28) | 316  5.27  1.40 (1.19-1.66) | 591  2.28  1.04 (0.93-1.16) | 467  2.96  1.30 (1.14-1.49) | 550  3.31  1.01 (0.90-1.14) |
| 2008 | 1012  3.11  1.12 (1.02-1.22) | 88  17.51  0.78 (0.59-1.02) | 271  4.49  1.20 (1.00-1.43) | 653  2.51  1.14 (1.02-1.28) | 458  2.89  1.27 (1.11-1.46) | 554  3.32  1.02 (0.90-1.14) |
| 2009 | 1169  3.60  1.29 (1.19-1.41) | 98  20.18  0.90 (0.68-1.17) | 336  5.62  1.50 (1.27-1.77) | 735  2.83  1.29 (1.15-1.43) | 489  3.09  1.36 (1.19-1.56) | 680  4.08  1.25 (1.12-1.39) |
| 2010 | 1249  3.86  1.39 (1.27-1.51) | 128  26.02  1.15 (0.90-1.48) | 308  5.23  1.39 (1.18-1.65) | 813  3.13  1.42 (1.28-1.58) | 544  3.45  1.52 (1.33-1.74) | 705  4.24  1.30 (1.16-1.45) |
| 2011 | 1204  3.76  1.35 (1.24-1.47) | 107  23.32  1.03 (0.80-1.35) | 248  4.32  1.15 (0.96-1.38) | 849  3.28  1.49 (1.35-1.66) | 562  3.60  1.59 (1.39-1.81) | 642  3.90  1.19 (1.07-1.34) |
| 2012 | 1185  3.77  1.35 (1.24-1.47) | 125  27.90  1.24 (0.96-1.59) | 282  5.20  1.38 (1.16-1.65) | 778  3.04  1.38 (1.24-1.54) | 569  3.71  1.64 (1.44-1.86) | 616  3.82  1.17 (1.04-1.31) |
| 2013 | 1153  3.76  1.35 (1.24-1.47) | 110  25.42  1.13 (0.87-1.46) | 264  4.95  1.32 (1.11-1.57) | 779  3.13  1.42 (1.28-1.58) | 534  3.57  1.57 (1.38-1.80) | 619  3.94  1.20 (1.07-1.35) |
| 2014 | 1112  3.72  1.34 (1.23-1.46) | 111  25.26  1.12 (0.86-1.45) | 260  4.98  1.33 (1.11-1.59) | 741  3.06  1.39 (1.25-1.55) | 520  3.57  1.57 (1.38-1.80) | 592  3.87  1.18 (1.05-1.33) |
| 2015 | 938  3.21  1.15 (1.05-1.26) | 91  20.41  0.91 (0.69-1.19) | 237  4.48  1.19 (1.00-1.43) | 610  2.60  1.18 (1.06-1.32) | 439  3.08  1.36 (1.18-1.56) | 499  3.33  1.02 (0.90-1.15) |
| 2016 | 951  3.28  1.18 (1.07-1.29) | 132  28.01  1.24 (0.97-1.60) | 230  4.15  1.11 (0.92-1.33) | 589  2.56  1.16 (1.04-1.30) | 419  2.97  1.31 (1.14-1.50) | 532  3.57  1.09 (0.97-1.23) |
| 2017 | 972  3.31  1.19 (1.09-1.30) | 133  27.91  1.24 (0.96-1.59) | 243  4.25  1.13 (0.95-1.36) | 596  2.57  1.17 (1.05-1.31) | 427  2.99  1.32 (1.15-1.52) | 545  3.61  1.10 (0.98-1.24) |
| 2018 | 1068  3.59  1.29 (1.18-1.41) | 144  30.23  1.34 (1.05-1.71) | 230  3.98  1.06 (0.88-1.27) | 694  2.95  1.34 (1.20-1.50) | 481  3.32  1.46 (1.28-1.68) | 587  3.84  1.17 (1.05-1.32) |
| Total | 22,944  3.61 | 2678  27.76 | 5680  4.88 | 14,586  2.86 | 9988  3.22 | 12,956  3.97 |

Each cell contains: (1) the number of events, (2) the incidence rate (per 1000 person-years), and (3) the incidence rate ratios (IRR) with 95% Confidence intervals (CI) compared to the reference year 1999. Left-hand side of table: The totals of (1)-(3) are presented by calendar year. Right-hand side of table: For each calendar year (1)-(3) are presented by age group and sex.

**Table E in S1 File. General anaesthesia records in medical specialist practice in 0–5-year-old Danish children, 1999-2018.**

|  | Events  Rate (per 1000)  IRR (95% CI) vs. 1999 | **Sex** | | **Chronic disease** | |
| --- | --- | --- | --- | --- | --- |
| **Year** | **Total** | **Female** | **Male** | **No** | **Yes** |
| 1999 | 25,186  74.07  Ref. year for IRR | 10,276  62.16 | 14,910  85.33 | 24,087  74.41 | 1099  110.50 |
| 2000 | 26,832  79.72  1.08 (1.06-1.09) | 10,931  66.82  1.07 (1.05-1.10) | 15,901  91.91  1.08 (1.05-1.10) | 25,700  80.29  1.08 (1.06-1.10) | 1132  113.80  1.03 (0.95-1.12) |
| 2001 | 27,728  82.75  1.12 (1.10-1.14) | 11,251  69.00  1.11 (1.08-1.14) | 16,477  95.80  1.12 (1.10-1.15) | 26,554  83.39  1.12 (1.10-1.14) | 1174  118.22  1.07 (0.99-1.16) |
| 2002 | 28,688  86.49  1.17 (1.15-1.19) | 11,582  71.68  1.15 (1.12-1.18) | 17,106  100.57  1.18 (1.15-1.20) | 27,546  87.38  1.17 (1.15-1.19) | 1142  115.74  1.05 (0.96-1.14) |
| 2003 | 28,816  87.51  1.18 (1.16-1.20) | 11,659  72.68  1.17 (1.14-1.20) | 17,157  101.59  1.19 (1.16-1.22) | 27,597  88.20  1.19 (1.16-1.21) | 1219  123.06  1.11 (1.03-1.21) |
| 2004 | 31,264  95.41  1.29 (1.27-1.31) | 12,784  80.09  1.29 (1.26-1.32) | 18,480  109.97  1.29 (1.26-1.32) | 29,925  96.13  1.29 (1.27-1.31) | 1339  135.73  1.23 (1.13-1.33) |
| 2005 | 27,852  85.45  1.15 (1.13-1.17) | 11,437  72.01  1.16 (1.13-1.19) | 16,415  98.22  1.15 (1.13-1.18) | 26,641  86.11  1.16 (1.14-1.18) | 1211  121.38  1.10 (1.01-1.19) |
| 2006 | 29,520  91.09  1.23 (1.21-1.25) | 11,995  75.91  1.22 (1.19-1.25) | 17,525  105.53  1.24 (1.21-1.26) | 28,256  91.98  1.24 (1.21-1.26) | 1264  124.39  1.13 (1.04-1.22) |
| 2007 | 31,819  98.19  1.33 (1.30-1.35) | 13,131  83.16  1.34 (1.30-1.37) | 18,688  112.48  1.32 (1.29-1.35) | 30,443  99.21  1.33 (1.31-1.36) | 1376  133.55  1.21 (1.12-1.31) |
| 2008 | 30,493  93.83  1.27 (1.25-1.29) | 12,310  77.69  1.25 (1.22-1.28) | 18,183  109.19  1.28 (1.25-1.31) | 29,147  94.77  1.27 (1.25-1.30) | 1346  128.53  1.16 (1.07-1.26) |
| 2009 | 30,541  94.11  1.27 (1.25-1.29) | 12,521  79.22  1.27 (1.24-1.31) | 18,020  108.25  1.27 (1.24-1.30) | 29,178  95.02  1.28 (1.26-1.30) | 1363  126.86  1.15 (1.06-1.24) |
| 2010 | 33,884  104.77  1.41 (1.39-1.44) | 13,838  87.89  1.41 (1.38-1.45) | 20,046  120.78  1.42 (1.39-1.45) | 32,280  105.55  1.42 (1.39-1.44) | 1604  143.99  1.30 (1.21-1.41) |
| 2011 | 32,411  101.16  1.37 (1.34-1.39) | 13,263  84.96  1.37 (1.33-1.40) | 19,148  116.55  1.37 (1.34-1.40) | 30,764  101.72  1.37 (1.34-1.39) | 1647  142.81  1.29 (1.20-1.39) |
| 2012 | 31,556  100.43  1.36 (1.33-1.38) | 13,039  85.14  1.37 (1.33-1.41) | 18,517  114.97  1.35 (1.32-1.38) | 29,808  100.81  1.35 (1.33-1.38) | 1748  146.15  1.32 (1.23-1.43) |
| 2013 | 28,748  93.79  1.27 (1.25-1.29) | 11,730  78.52  1.26 (1.23-1.30) | 17,018  108.31  1.27 (1.24-1.30) | 27,049  94.07  1.26 (1.24-1.29) | 1699  137.14  1.24 (1.15-1.34) |
| 2014 | 28,329  94.91  1.28 (1.26-1.30) | 11,628  79.85  1.28 (1.25-1.32) | 16,701  109.25  1.28 (1.25-1.31) | 26,628  95.33  1.28 (1.26-1.30) | 1701  134.97  1.22 (1.13-1.32) |
| 2015 | 26,972  92.36  1.25 (1.23-1.27) | 10,929  76.74  1.23 (1.20-1.27) | 16,043  107.22  1.26 (1.23-1.28) | 25,178  92.33  1.24 (1.22-1.26) | 1794  137.74  1.25 (1.16-1.34) |
| 2016 | 26,332  90.74  1.23 (1.20-1.25) | 10,567  74.81  1.20 (1.17-1.24) | 15,765  105.84  1.24 (1.21-1.27) | 24,511  90.56  1.22 (1.20-1.24) | 1821  135.88  1.23 (1.14-1.33) |
| 2017 | 27,882  95.04  1.28 (1.26-1.31) | 11,220  78.69  1.27 (1.23-1.30) | 16,662  110.57  1.30 (1.27-1.32) | 25,881  94.67  1.27 (1.25-1.29) | 2001  144.76  1.31 (1.22-1.41) |
| 2018 | 27,193  91.35  1.23 (1.21-1.25) | 11,000  76.03  1.22 (1.19-1.26) | 16,193  106.01  1.24 (1.22-1.27) | 25,154  90.82  1.22 (1.20-1.24) | 2039  141.98  1.28 (1.19-1.38) |
| Total | 582,046  93.43 | 237,091  76.54 | 344,955  105.74 | 552,327  91.99 | 29,719  131.88 |

Each cell contains: (1) the number of events, (2) the incidence rate (per 1000 person-years), and (3) the incidence rate ratios (IRR) with 95% Confidence intervals (CI) compared to the reference year 1999. Left-hand side of table: The totals of (1)-(3) are presented by calendar year. Right-hand side of table: For each calendar year (1)-(3) are presented by sex and chronic disease status.

**Table F in S1 File. Top 5 most frequent procedure codes in Danish children, 0-5 years of age, 1999-2018, by surgical specialty.**

| **Surgical specialty** | **Procedure name and NOMESCO code** | **Frequency (% within surgical specialty)** |
| --- | --- | --- |
| Otorhinolaryngology (ENT) | Otomicroscopy, KUDB22 | 8706 (14.0%) |
|  | Adenotonsillectomy, KEMB20 | 7620 (12.3%) |
|  | Grommets, KDCA20 | 7312 (11.8%) |
|  | Paracentesis of tympanic membrane, KDCA10 | 5508 (8.9%) |
|  | Frenectomy of tongue, KEJC20 | 4480 (7.2%) |
| Paediatric surgery | Inguinal hernia repair, KJAB00 | 7211 (16.7%) |
|  | Operation for undescended or ectopic testis, KFH00 | 2222 (5.1%) |
|  | Correction of hypospadia, KGH60 | 2031 (4.7%) |
|  | Gastroscopic biopsy, KUJD05 | 1615 (3.7%) |
|  | Endoscopic dilatation of oesophagus, KJCA55 | 1509 (3.5%) |
| Minor surgical procedures | Lumbar puncture, KTAB00 | 7876 (34.3%) |
|  | Other minor surgical procedure, KTPW10 | 1989 (8.7%) |
|  | Percutaneous puncture of bladder, KTKC10 | 1566 (6.8%) |
|  | Catheterisation of right atrium or ventricle, KTFC00 | 1247 (5.4%) |
|  | Retrograde catheterisation of left atrium or ventricle, KTFC10B | 565 (2.5%) |
| Orthopaedic surgery | Internal fixation of humerus fracture, KNBJ41 | 2350 (10.9%) |
|  | Closed reposition of fracture of ulna and radius, KNCJ06 | 1560 (7.3%) |
|  | Removal of internal fixation from elbow or forearm, KNCU49 | 1132 (5.3%) |
|  | Forcible manipulation of ankle or foot joint, KNHT19 | 1086 (5.1%) |
|  | Correction of ankle or foot deformity, KNHT39 | 820 (3.8%) |
| Cardiothoracic surgery | Intercostal insertion of pleural drainage tube, KGAA10 | 1817 (15.2%) |
|  | Flexible bronchoscopy, KUGC12 | 1647 (13.7%) |
|  | Rigid bronchoscopy, KUGC02 | 643 (5.4%) |
|  | Other total cardiopulmonary bypass at concurrent surgical procedure, KFXA96 | 595 (5.0%) |
|  | Ductus arteriosus closure, KFDE32 | 463 (3.9%) |
| Plastic surgery | Cleft palate repair, KEHC30 | 1842 (15.8%) |
|  | Correction of cleft lip, KEAB30 | 1417 (12.2%) |
|  | Incision of skin of trunk, KQBA10 | 747 (6.4%) |
|  | Major dressing of wound of skin of trunk, KQBB10 | 706 (6.1%) |
|  | Suture of skin of upper limb, KQCB00 | 406 (3.5%) |
| Neurosurgery | Shunt revision, KAAF20 | 850 (17.9%) |
|  | Ventriculoperitoneal shunt, KAAF05 | 712 (15.0%) |
|  | Craniofacial reconstruction in congenital malformations, KAAK30 | 414 (8.7%) |
|  | Craniosynostosis repair, KAAK20 | 239 (5.0%) |
|  | Extirpation of intracranial lesion, KAAB00 | 168 (3.5%) |
| Ophthalmology (eye) | Other operation on eyeball, KCDW99 | 485 (10.3%) |
|  | Recession and resection of extraocular muscle, KCEC10 | 399 (8.5%) |
|  | Lacrimal duct dilatation, KCCC40 | 359 (7.7%) |
|  | Recession of extraocular muscle, KCEC00 | 319 (6.8%) |
|  | Removal of foreign body from cornea, KCGC10 | 311 (6.6%) |
| Vascular surgery | Excision of cervical lymph nodes, KPJD41 | 407 (38.0%) |
|  | Incision of lymph node, KPJW10 | 265 (24.7%) |
|  | Excision of other lymph nodes, KPJD99 | 49 (4.6%) |
|  | Injection of therapeutic agent into or percutaneous occlusion of other vein, KPHT99 | 41 (3.8%) |
|  | Insertion of stent into other vein, KPHQ99 | 33 (3.1%) |
| Oral/maxillofacial surgery | Extraction of tooth, KEBA00 | 509 (54.0%) |
|  | Surgical removal of tooth, KEBA10 | 64 (6.8%) |
|  | Other operation on tooth, KEBW99 | 56 (5.9%) |
|  | Suture of gingiva, KECB00 | 39 (4.1%) |
|  | Other excision of maxilla, KEEB99 | 35 (3.7%) |
| Gynaecology | Photocolposcopy, KULD02A | 79 (13.1%) |
|  | Vaginoscopy, KULD12 | 65 (10.7%) |
|  | Colposcopy, KULD02 | 46 (7.6%) |
|  | Culdoscopy, KLEA01 | 43 (7.1%) |
|  | Incision of vulva or perineum, KLFA00 | 35 (5.8%) |

**Table G in S1 File. Inpatient surgical procedures by specialties in Danish children with chronic disease, 0-5 years of age, 1999-2018.**

|  | Events  Rate (per 1000)  IRR (95% CI) vs. 1999 | **Surgical specialties** | | | |
| --- | --- | --- | --- | --- | --- |
| **Year** | **All surgeries** | **Paediatric** | **Minor** | **Orthopaedic** | **Cardiothoracic** |
| 1999 | 1545  155.34  Ref. year for IRR | 368  37.00 | 280  28.15 | 143  14.38 | 189  19.00 |
| 2000 | 1569  157.74  1.02 (0.95-1.09) | 449  45.14  1.22 (1.06-1.40) | 220  22.12  0.79 (0.66-0.94) | 127  12.77  0.89 (0.70-1.13) | 159  15.98  0.84 (0.68-1.04) |
| 2001 | 1794  180.65  1.16 (1.09-1.24) | 491  49.44  1.34 (1.17-1.53) | 328  33.03  1.17 (1.00-1.38) | 132  13.29  0.92 (0.73-1.17) | 196  19.74  1.04 (0.85-1.27) |
| 2002 | 1762  178.57  1.15 (1.07-1.23) | 479  48.54  1.31 (1.15-1.50) | 351  35.57  1.26 (1.08-1.48) | 116  11.76  0.82 (0.64-1.04) | 214  21.69  1.14 (0.94-1.39) |
| 2003 | 1670  168.59  1.09 (1.01-1.16) | 454  45.83  1.24 (1.08-1.42) | 343  34.63  1.23 (1.05-1.44) | 108  10.90  0.76 (0.59-0.97) | 165  16.66  0.88 (0.71-1.08) |
| 2004 | 1766  179.01  1.15 (1.08-1.23) | 431  43.69  1.18 (1.03-1.36) | 380  38.52  1.37 (1.17-1.60) | 121  12.26  0.85 (0.67-1.09) | 239  24.23  1.27 (1.05-1.54) |
| 2005 | 1723  172.69  1.11 (1.04-1.19) | 406  40.69  1.10 (0.96-1.27) | 334  33.48  1.19 (1.01-1.39) | 109  10.92  0.76 (0.59-0.97) | 206  20.65  1.09 (0.89-1.32) |
| 2006 | 1791  176.26  1.13 (1.06-1.21) | 457  44.97  1.22 (1.06-1.39) | 354  34.84  1.24 (1.06-1.45) | 103  10.14  0.71 (0.55-0.91) | 242  23.82  1.25 (1.04-1.52) |
| 2007 | 1583  153.65  0.99 (0.92-1.06) | 431  41.83  1.13 (0.98-1.30) | 221  21.45  0.76 (0.64-0.91) | 101  9.80  0.68 (0.53-0.88) | 209  20.29  1.07 (0.88-1.30) |
| 2008 | 1410  134.64  0.87 (0.81-0.93) | 391  37.34  1.01 (0.88-1.16) | 224  21.39  0.76 (0.64-0.91) | 87  8.31  0.58 (0.44-0.75) | 168  16.04  0.84 (0.69-1.04) |
| 2009 | 1574  146.50  0.94 (0.88-1.01) | 472  43.93  1.19 (1.04-1.36) | 247  22.99  0.82 (0.69-0.97) | 97  9.03  0.63 (0.49-0.81) | 155  14.43  0.76 (0.61-0.94) |
| 2010 | 1697  152.34  0.98 (0.92-1.05) | 512  45.96  1.24 (1.09-1.42) | 281  25.23  0.90 (0.76-1.06) | 114  10.23  0.71 (0.56-0.91) | 159  14.27  0.75 (0.61-0.93) |
| 2011 | 1663  144.20  0.93 (0.87-0.99) | 481  41.71  1.13 (0.98-1.29) | 311  26.97  0.96 (0.82-1.13) | 120  10.41  0.72 (0.57-0.92) | 149  12.92  0.68 (0.55-0.84) |
| 2012 | 1643  137.37  0.88 (0.83-0.95) | 471  39.38  1.06 (0.93-1.22) | 281  23.49  0.83 (0.71-0.98) | 124  10.37  0.72 (0.57-0.92) | 167  13.96  0.73 (0.60-0.90) |
| 2013 | 1808  145.93  0.94 (0.88-1.01) | 567  45.77  1.24 (1.08-1.41) | 284  22.92  0.81 (0.69-0.96) | 122  9.85  0.68 (0.54-0.87) | 174  14.04  0.74 (0.60-0.91) |
| 2014 | 1793  142.27  0.92 (0.86-0.98) | 508  40.31  1.09 (0.95-1.25) | 328  26.03  0.92 (0.79-1.08) | 117  9.28  0.65 (0.51-0.82) | 162  12.85  0.68 (0.55-0.83) |
| 2015 | 1564  120.08  0.77 (0.72-0.83) | 500  38.39  1.04 (0.91-1.19) | 277  21.27  0.76 (0.64-0.89) | 100  7.68  0.53 (0.41-0.69) | 136  10.44  0.55 (0.44-0.68) |
| 2016 | 1564  116.70  0.75 (0.70-0.81) | 530  39.55  1.07 (0.94-1.22) | 260  19.40  0.69 (0.58-0.82) | 86  6.42  0.45 (0.34-0.58) | 143  10.67  0.56 (0.45-0.70) |
| 2017 | 1583  114.52  0.74 (0.69-0.79) | 552  39.93  1.08 (0.95-1.23) | 245  17.72  0.63 (0.53-0.75) | 94  6.80  0.47 (0.36-61) | 164  11.86  0.62 (0.51-0.77) |
| 2018 | 1630  113.50  0.73 (0.68-0.78) | 477  33.22  0.90 (0.78-1.03) | 242  16.85  0.60 (0.50-0.71) | 99  6.89  0.48 (0.37-0.62) | 182  12.67  0.67 (0.54-0.82) |
| Total | 33,132  147.02 | 9427  41.83 | 5791  25.70 | 2220  9.85 | 3578  15.88 |

Each cell contains: (1) the number of events, (2) the incidence rate (per 1000 person-years), and (3) the incidence rate ratios (IRR) with 95% Confidence intervals (CI) compared to the reference year 1999. Left-hand side of table: The totals of (1)-(3) are presented by calendar year. Right-hand side of table: For each calendar year (1)-(3) are presented by four different surgical specialties.

**Table H in S1 File. Inpatient surgical procedures by specialties in Danish children with chronic disease, 0-5 years of age, 1999-2018.**

|  | Events  Rate (per 1000)  IRR (95% CI) vs. 1999 | **Surgical specialties** | | | |
| --- | --- | --- | --- | --- | --- |
| **Year** | **All surgeries** | **Otorhinolaryngology** | **Plastic** | **Neurological** | **Eye** |
| 1999 | 1545  155.34  Ref. year for IRR | 363  36.50 | 154  15.48 | 106  10.66 | 48  4.83 |
| 2000 | 1569  157.74  1.02 (0.95-1.09) | 391  39.31  1.08 (0.93-1.24) | 160  16.09  1.04 (0.83-1.30) | 123  12.37  1.16 (0.89-1.50) | 36  3.62  0.75 (0.49-1.16) |
| 2001 | 1794  180.65  1.16 (1.09-1.24) | 427  43.00  1.18 (1.02-1.36) | 163  16.41  1.06 (0.85-1.32) | 117  11.78  1.11 (0.85-1.44) | 45  4.53  0.94 (0.63-1.41) |
| 2002 | 1762  178.57  1.15 (1.07-1.23) | 426  43.17  1.18 (1.03-1.36) | 169  17.13  1.11 (0.89-1.38) | 98  9.93  0.93 (0.71-1.23) | 37  3.75  0.78 (0.51-1.19) |
| 2003 | 1670  168.59  1.09 (1.01-1.16) | 434  43.81  1.20 (1.04-1.38) | 120  12.11  0.78 (0.62-0.99) | 113  11.41  1.07 (0.82-1.40) | 38  3.84  0.79 (0.52-1.22) |
| 2004 | 1766  179.01  1.15 (1.08-1.23) | 473  47.94  1.31 (1.15-1.51) | 149  15.10  0.98 (0.78-1.22) | 78  7.91  0.74 (0.55-0.99) | 26  2.64  0.55 (0.34-0.88) |
| 2005 | 1723  172.69  1.11 (1.04-1.19) | 503  50.41  1.38 (1.21-1.58) | 164  16.44  1.06 (0.85-1.32) | 99  9.92  0.93 (0.71-1.22) | 33  3.31  0.78 (0.66-0.91) |
| 2006 | 1791  176.26  1.13 (1.06-1.21) | 545  53.63  1.47 (1.29-1.68) | 150  14.76  0.95 (0.76-1.19) | 71  6.99  0.66 (0.49-0.89) | 26  2.56  0.53 (0.33-0.85) |
| 2007 | 1583  153.65  0.99 (0.92-1.06) | 465  45.13  1.24 (1.08-1.42) | 135  13.10  0.85 (0.67-1.07) | 93  9.03  0.85 (0.64-1.12) | 41  3.98  0.82 (0.54-1.25) |
| 2008 | 1410  134.64  0.87 (0.81-0.93) | 362  34.57  0.95 (0.82-1.10) | 131  12.51  0.81 (0.64-1.02) | 114  10.89  1.02 (0.78-1.33) | 32  3.06  0.63 (0.40-0.99) |
| 2009 | 1574  146.50  0.94 (0.88-1.01) | 460  42.81  1.17 (1.02-1.35) | 159  14.80  0.96 (0.77-1.19) | 62  5.77  0.54 (0.40-0.74) | 35  3.26  0.68 (0.44-1.04) |
| 2010 | 1697  152.34  0.98 (0.92-1.05) | 477  42.82  1.17 (1.02-1.34) | 155  13.91  0.90 (0.72-1.12) | 85  7.63  0.72 (0.54-0.95) | 37  3.32  0.69 (0.45-1.06) |
| 2011 | 1663  144.20  0.93 (0.87-0.99) | 419  36.33  1.00 (0.86-1.15) | 172  14.91  0.96 (0.77-1.20) | 86  7.46  0.70 (0.53-0.93) | 41  3.56  0.74 (0.49-1.12) |
| 2012 | 1643  137.37  0.88 (0.83-0.95) | 431  36.04  0.99 (0.86-1.14) | 177  14.80  0.96 (0.77-1.19) | 92  7.69  0.72 (0.55-0.95) | 22  1.84  0.38 (0.23-0.63) |
| 2013 | 1808  145.93  0.94 (0.88-1.01) | 484  39.07  1.07 (0.93-1.23) | 187  15.09  0.97 (0.79-1.21) | 95  7.67  0.72 (0.55-0.95) | 19  1.13  0.32 (0.19-0.54) |
| 2014 | 1793  142.27  0.92 (0.86-0.98) | 539  42.77  1.17 (1.03-1.34) | 146  11.58  0.75 (0.60-0.94) | 98  7.78  0.73 (0.55-0.96) | 33  2.62  0.54 (0.35-0.85) |
| 2015 | 1564  120.08  0.77 (0.72-0.83) | 437  33.55  0.92 (0.80-1.06) | 130  9.98  0.64 (0.51-0.81) | 69  5.30  0.50 (0.37-0.67) | 27  2.07  0.43 (0.27-0.69) |
| 2016 | 1564  116.70  0.75 (0.70-0.81) | 426  31.79  0.87 (0.76-1.00) | 144  10.74  0.69 (0.55-0.87) | 55  4.10  0.39 (0.28-0.53) | 27  2.01  0.42 (0.26-0.67) |
| 2017 | 1583  114.52  0.74 (0.69-0.79) | 388  28.07  0.77 (0.67-0.89) | 116  8.39  0.54 (0.43-0.69) | 85  6.15  0.58 (0.43-0.77) | 18  1.30  0.27 (0.16-0.46) |
| 2018 | 1630  113.50  0.73 (0.68-0.78) | 449  31.27  0.86 (0.75-0.98) | 169  11.77  0.76 (0.61-0.95) | 81  5.64  0.53 (0.40-0.71) | 34  2.37  0.49 (0.32-0.76) |
| Total | 33,132  147.02 | 8899  39.49 | 3050  13.53 | 1820  8.08 | 655  2.69 |

Each cell contains: (1) the number of events, (2) the incidence rate (per 1000 person-years), and (3) the incidence rate ratios (IRR) with 95% Confidence intervals (CI) compared to the reference year 1999. Left-hand side of table: The totals of (1)-(3) are presented by calendar year. Right-hand side of table: For each calendar year (1)-(3) are presented by four different surgical specialties.
