# Supplementary material for: Surgical procedures in Danish children 1999–2018
Source: PLoS One. 2023 Apr 27;18(4):e0285047. doi: 10.1371/journal.pone.0285047 (PMC10138199; doi:10.1371/journal.pone.0285047)

**Fig A in S2 File. Surgical procedures in private hospitals per 1000 person-years in Danish children 0-5 years of age, 1999-2018**

**
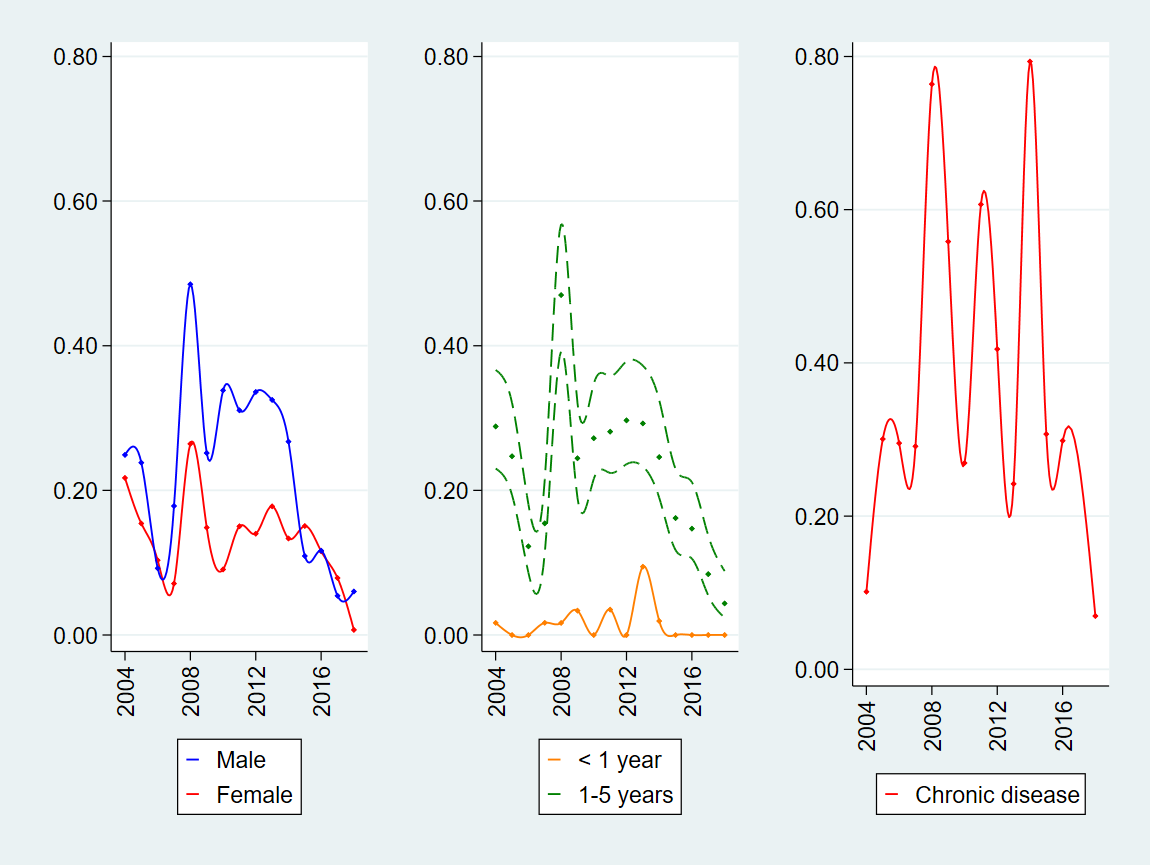
**

**Fig B in S2 File. Surgical procedures in public hospitals per 1000 person-years in Danish neonates, 1999-2018.** The upper curve represents all surgical procedures in neonates, the lower curve excludes tongue frenectomy. The absolute number of procedures are provided together with the whiskers for the 95% CI.
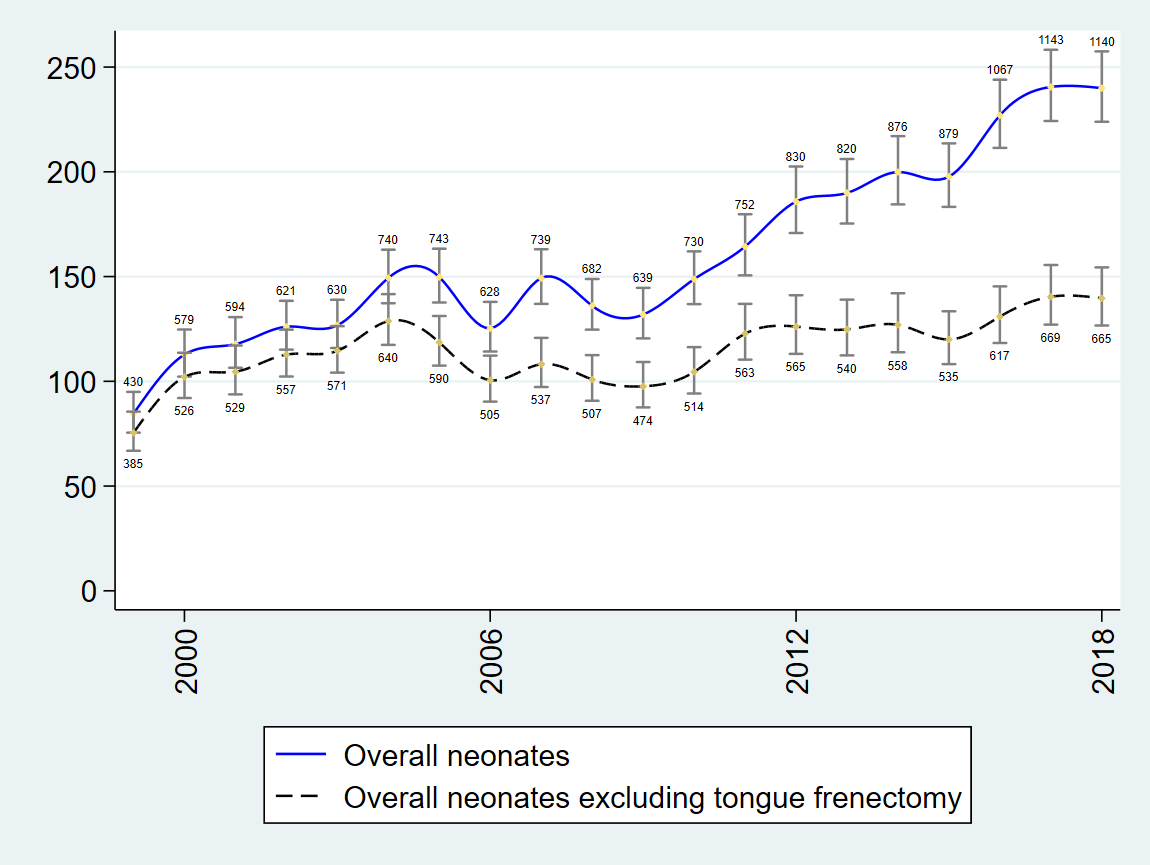


**Fig C in S2 File. Otorhinolaryngology (ENT) procedures in public hospitals per 1000 person-years in Danish neonates, 1999-2018.** The upper curve represents all surgical procedures in neonates, the lower curve excludes tongue frenectomy. The absolute number of procedures are provided together with the whiskers for the 95% CI.

**
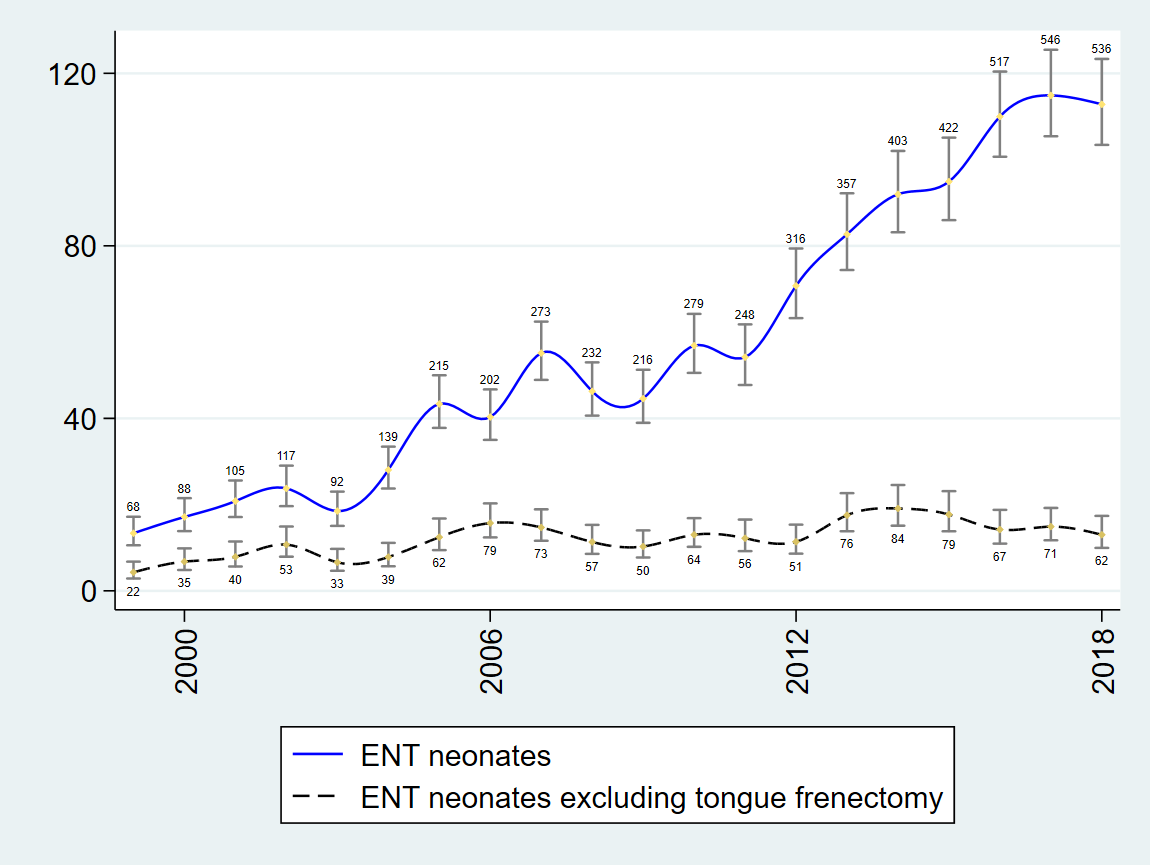
**

**Fig D in S2 File. Otorhinolaryngology (ENT) procedures per 1000 person-years in Danish children 0-5 years of age, 1999-2018.** Sex-specific incidence (left panel), age-specific incidence (middle panel) and chronic disease-specific incidence (right panel) with 95% pointwise confidence limits.


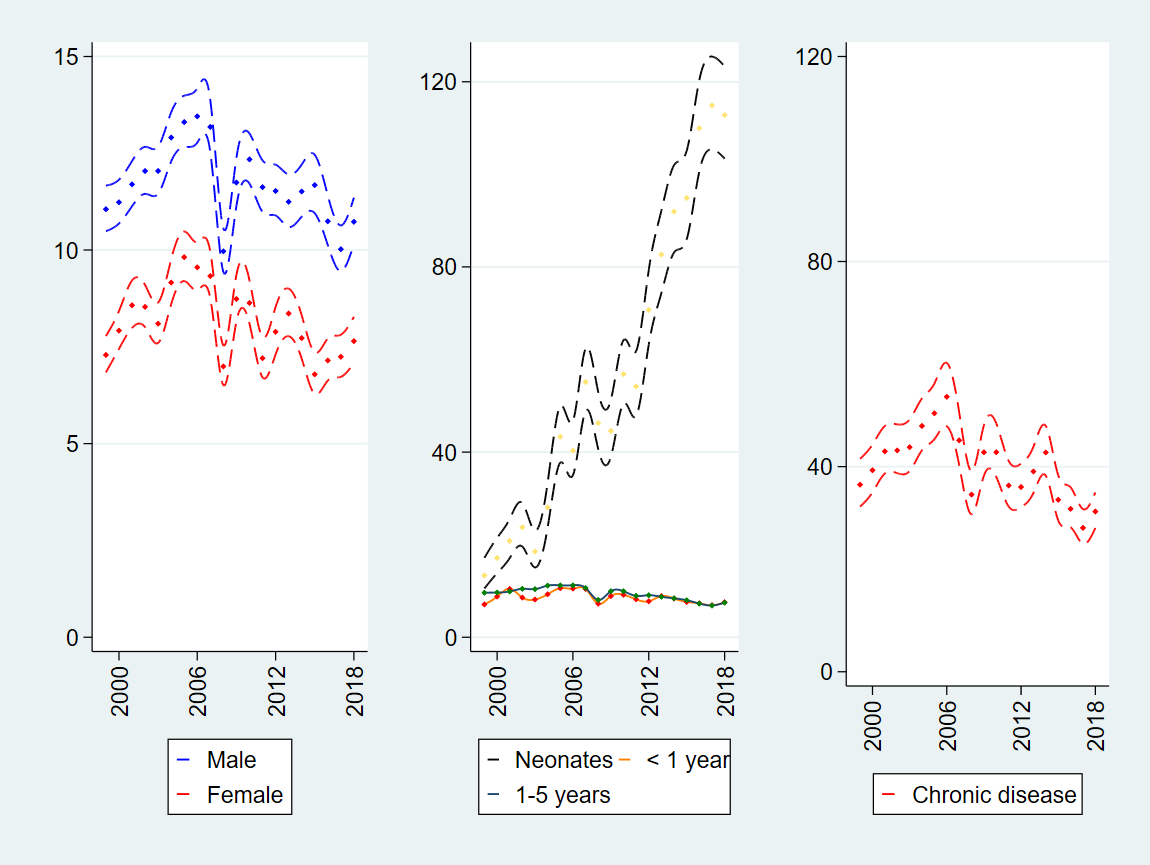


**Fig E in S2 File. Paediatric procedures per 1000 person-years in Danish children 0-5 years of age, 1999-2018.** Sex-specific incidence (left panel), age-specific incidence (middle panel) and chronic disease-specific incidence (right panel) with 95% pointwise confidence limits.


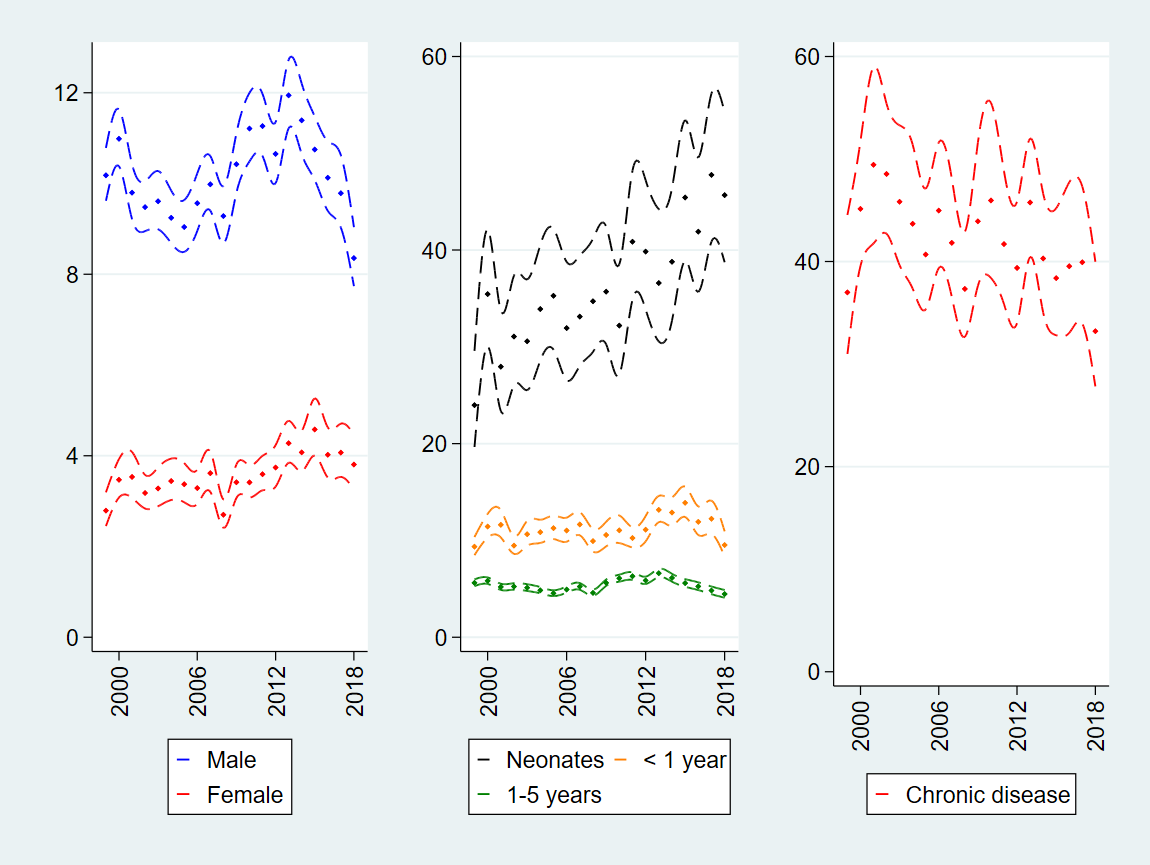


**Fig F in S2 File. Minor surgical procedures per 1000 person-years in Danish children 0-5 years of age, 1999-2018.** Sex-specific incidence (left panel), age-specific incidence (middle panel) and chronic disease-specific incidence (right panel) with 95% pointwise confidence limits.


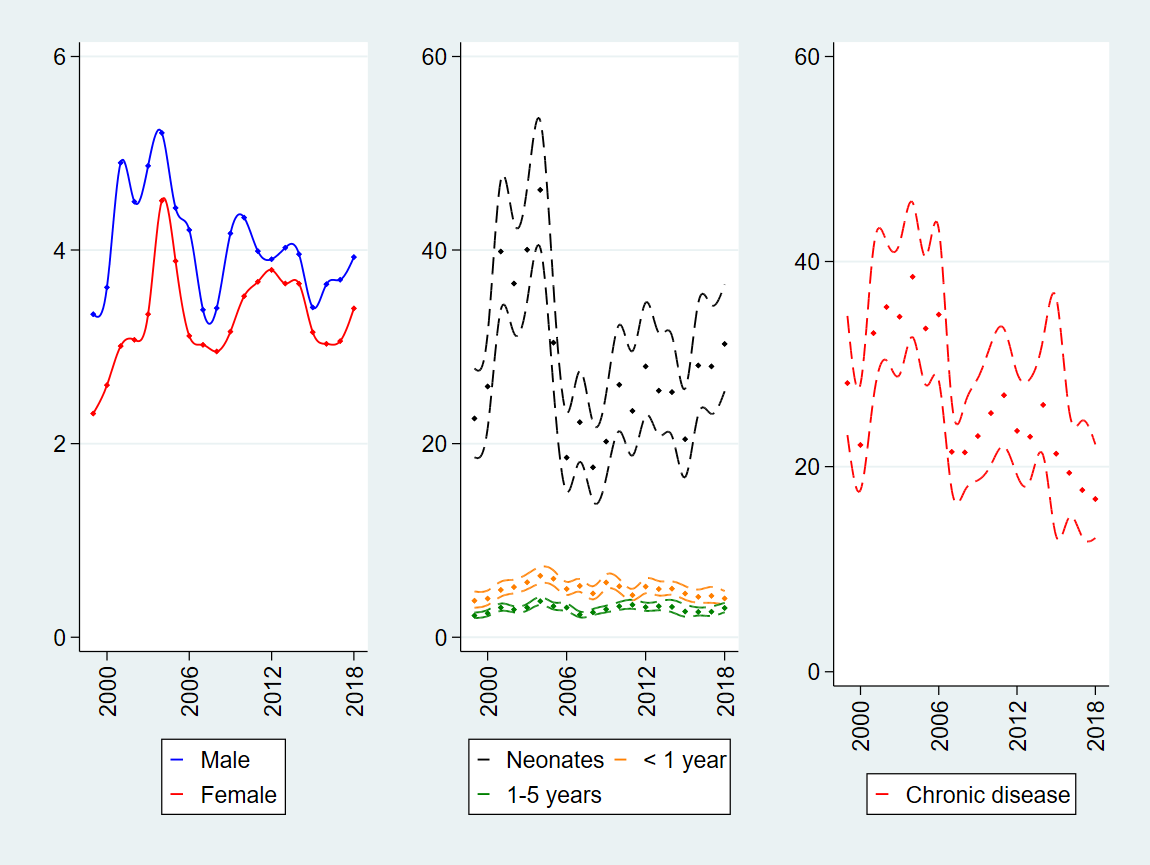


**Fig G in S2 File. Orthopaedic procedures per 1000 person-years in Danish children 0-5 years of age, 1999-2018.** Sex-specific incidence (left panel), age-specific incidence (middle panel) and chronic disease-specific incidence (right panel) with 95% pointwise confidence limits.


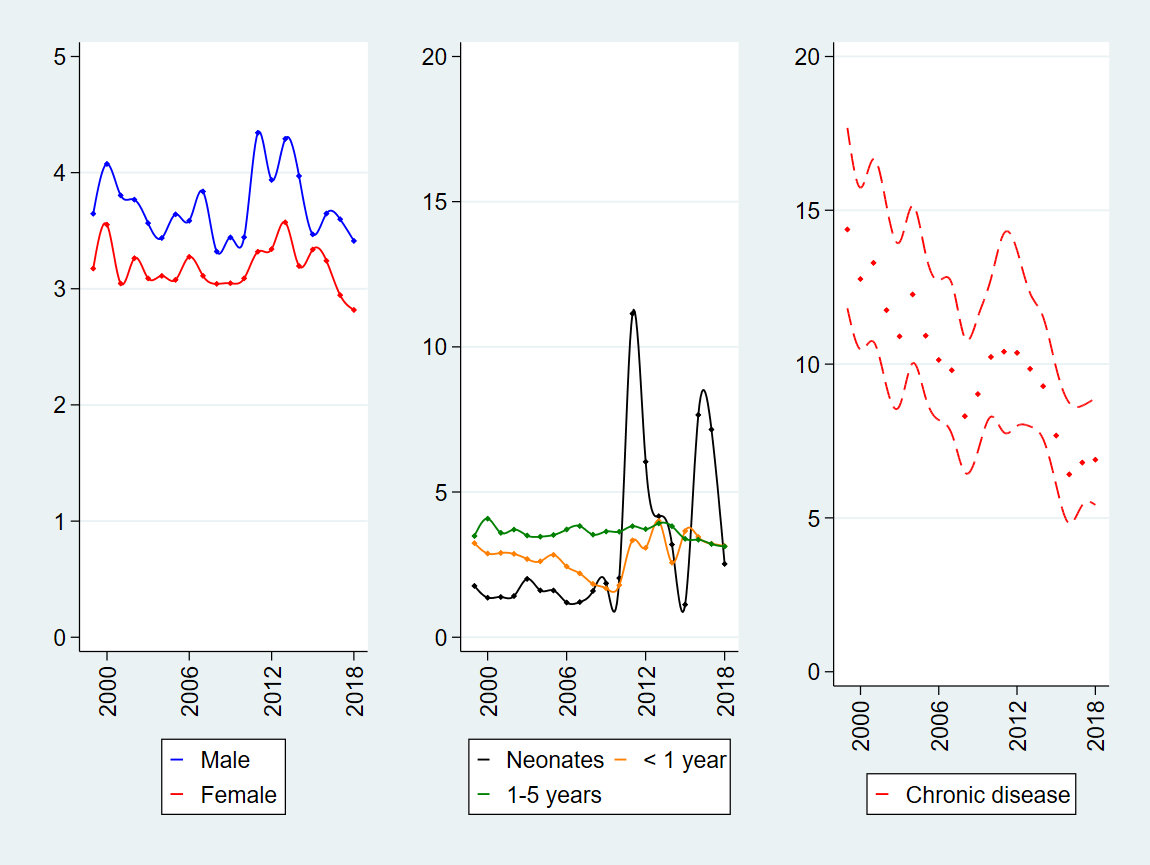


**Fig H in S2 File. Cardiothoracic procedures per 1000 person-years in Danish children 0-5 years of age, 1999-2018.** Sex-specific incidence (left panel), age-specific incidence (middle panel) and chronic disease-specific incidence (right panel) with 95% pointwise confidence limits.


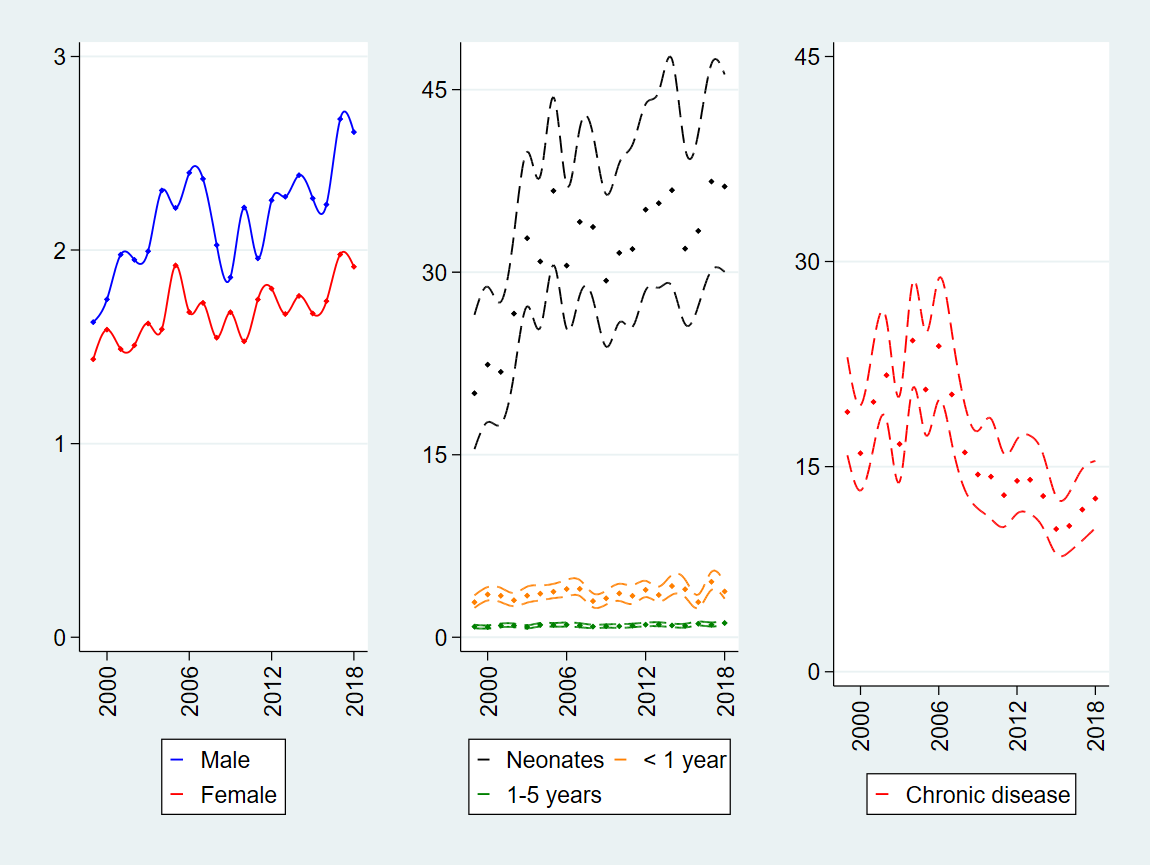


**Fig I in S2 File. Plastic surgery procedures per 1000 person-years in Danish children 0-5 years of age, 1999-2018.** Sex-specific incidence (left panel), age-specific incidence (middle panel) and chronic disease-specific incidence (right panel) with 95% pointwise confidence limits.


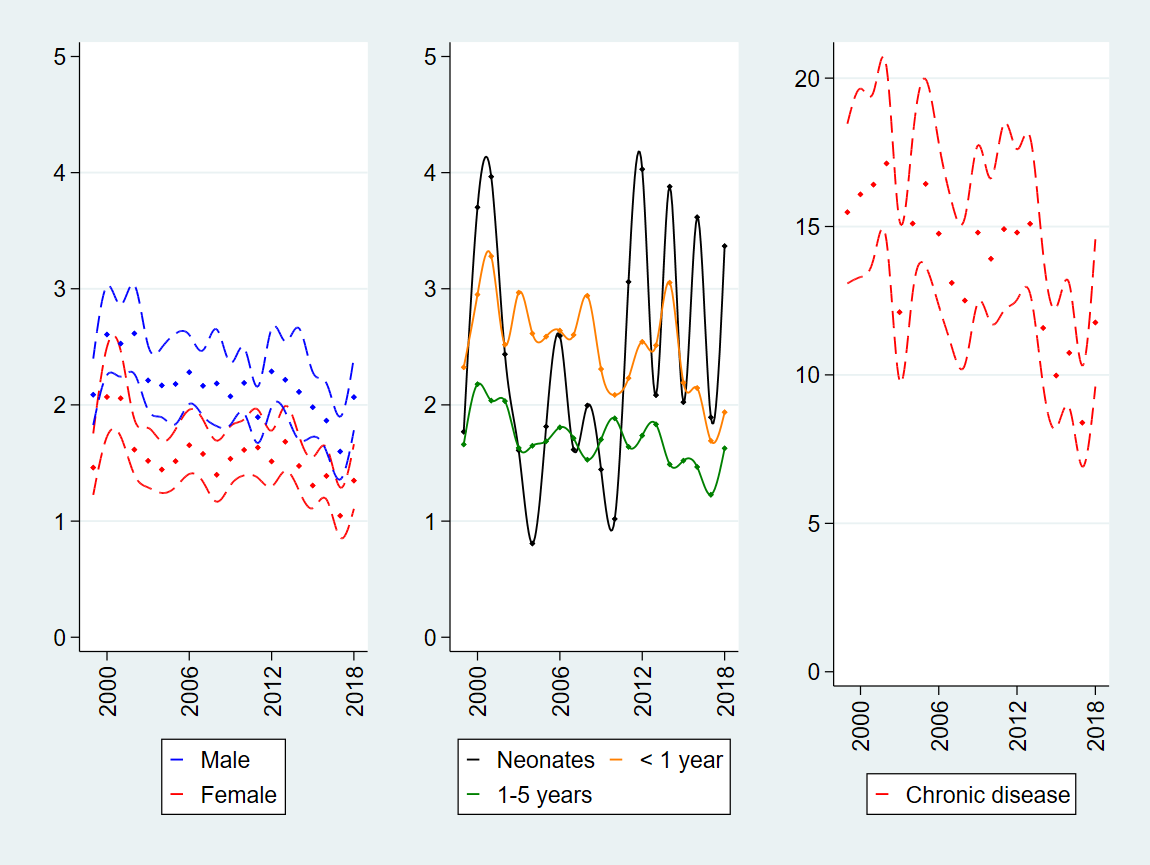


**Fig J in S2 File. Neurosurgical procedures per 1000 person-years in Danish children 0-5 years of age, 1999-2018.** Sex-specific incidence (left panel), age-specific incidence (middle panel) and chronic disease-specific incidence (right panel) with 95% pointwise confidence limits.


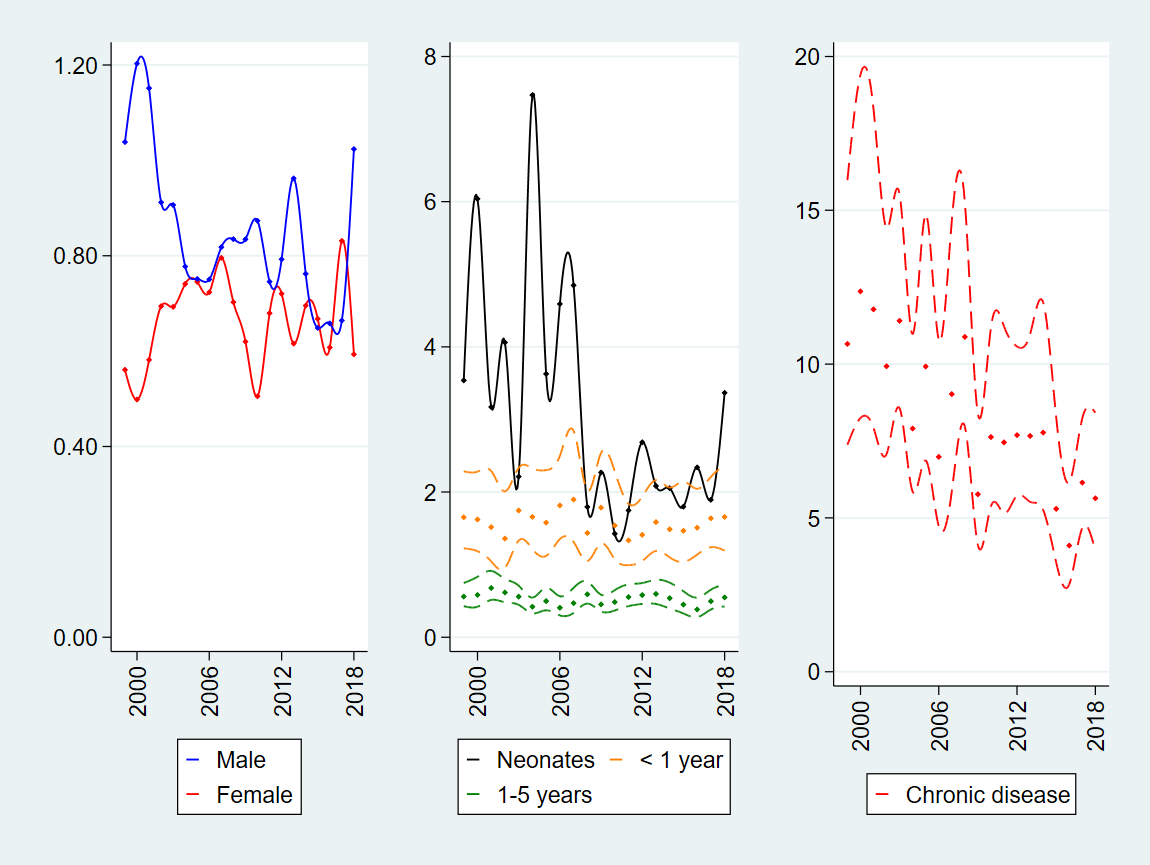


**Fig K in S2 File. Ophthalmology (eye) procedures per 1000 person-years in Danish children 0-5 years of age, 1999-2018.** Sex-specific incidence (left panel), age-specific incidence (middle panel) and chronic disease-specific incidence (right panel) with 95% pointwise confidence limits.


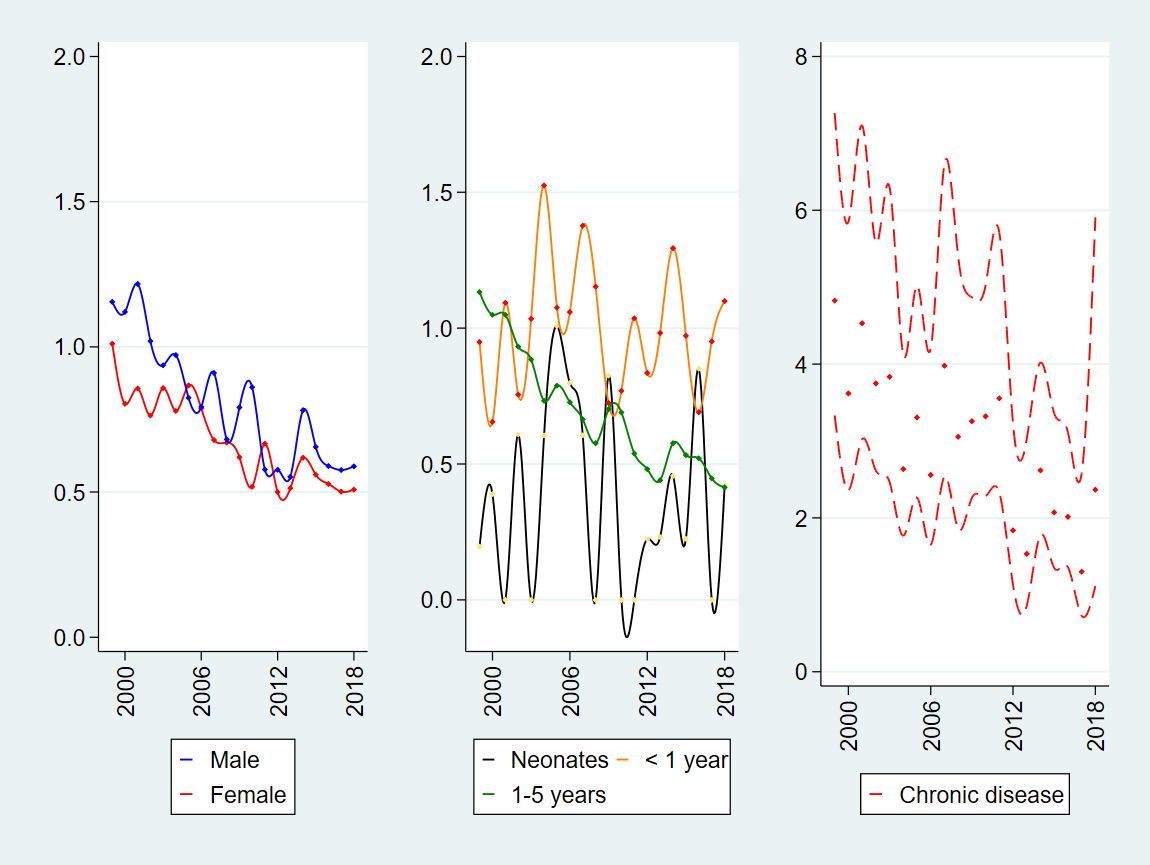


**Fig L in S2 File. Vascular procedures per 1000 person-years in Danish children 0-5 years of age, 1999-2018.** Sex-specific incidence (left panel), age-specific incidence (middle panel) and chronic disease-specific incidence (right panel) with 95% pointwise confidence limits.


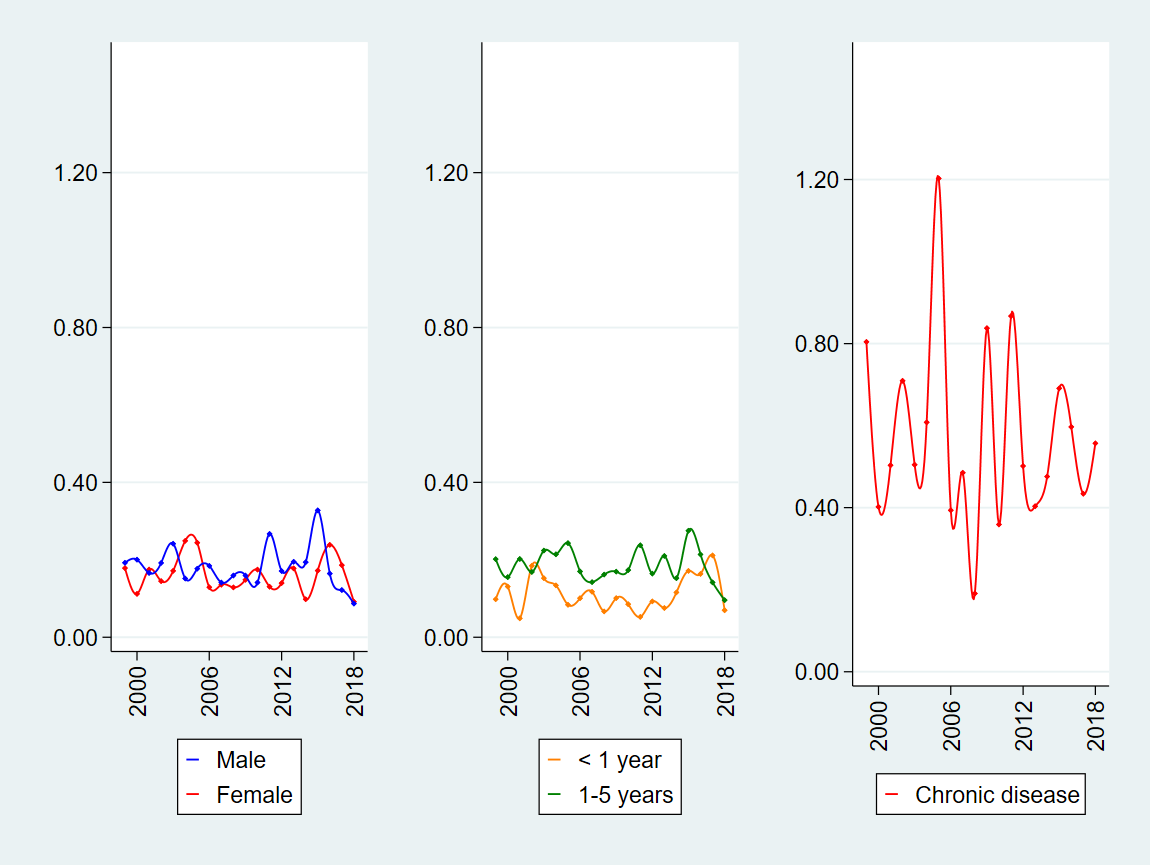


**Fig M in S2 File. Oral/maxillofacial procedures per 1000 person-years in Danish children 0-5 years of age, 1999-2018.** Sex-specific incidence (left panel), age-specific incidence (middle panel) and chronic disease-specific incidence (right panel) with 95% pointwise confidence limits.


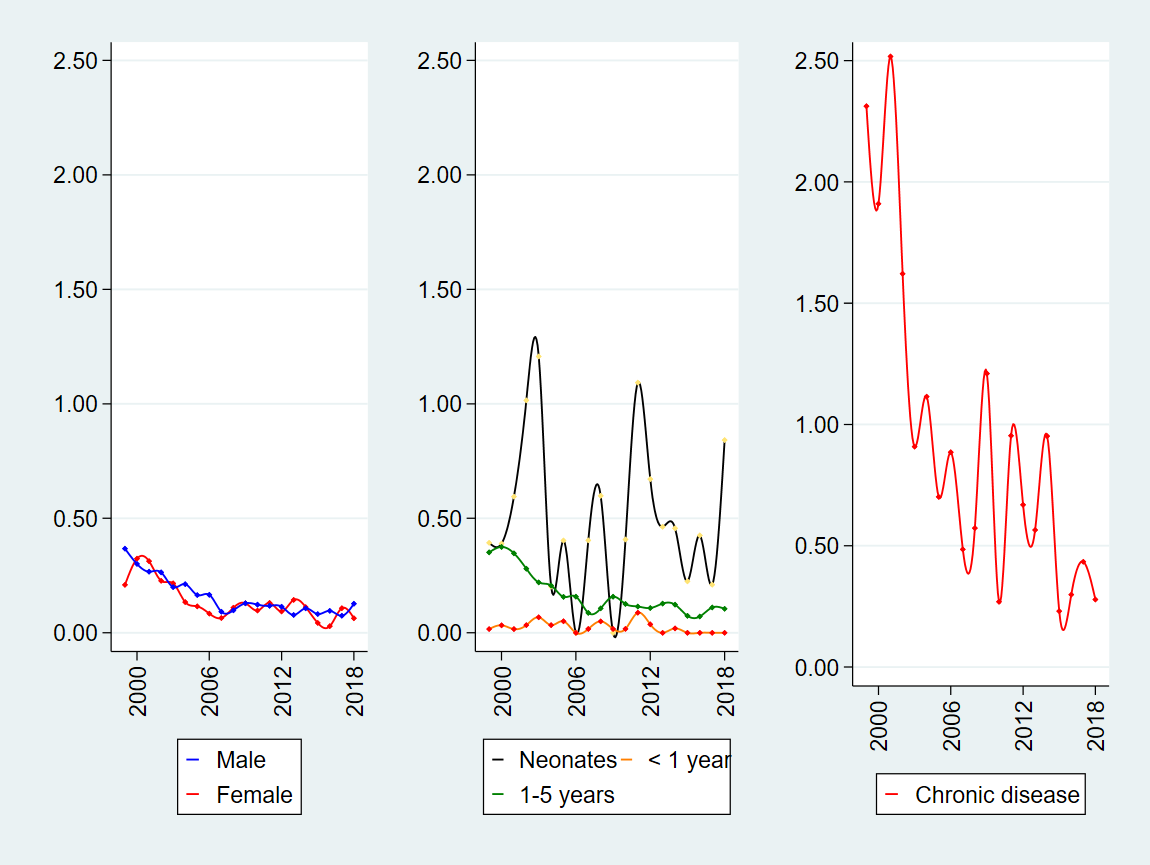


**Fig N in S2 File. Gynaecological procedures per 1000 person-years in Danish children 0-5 years of age, 1999-2018.** Sex-specific incidence (left panel), age-specific incidence (middle panel) and chronic disease-specific incidence (right panel) with 95% pointwise confidence limits.


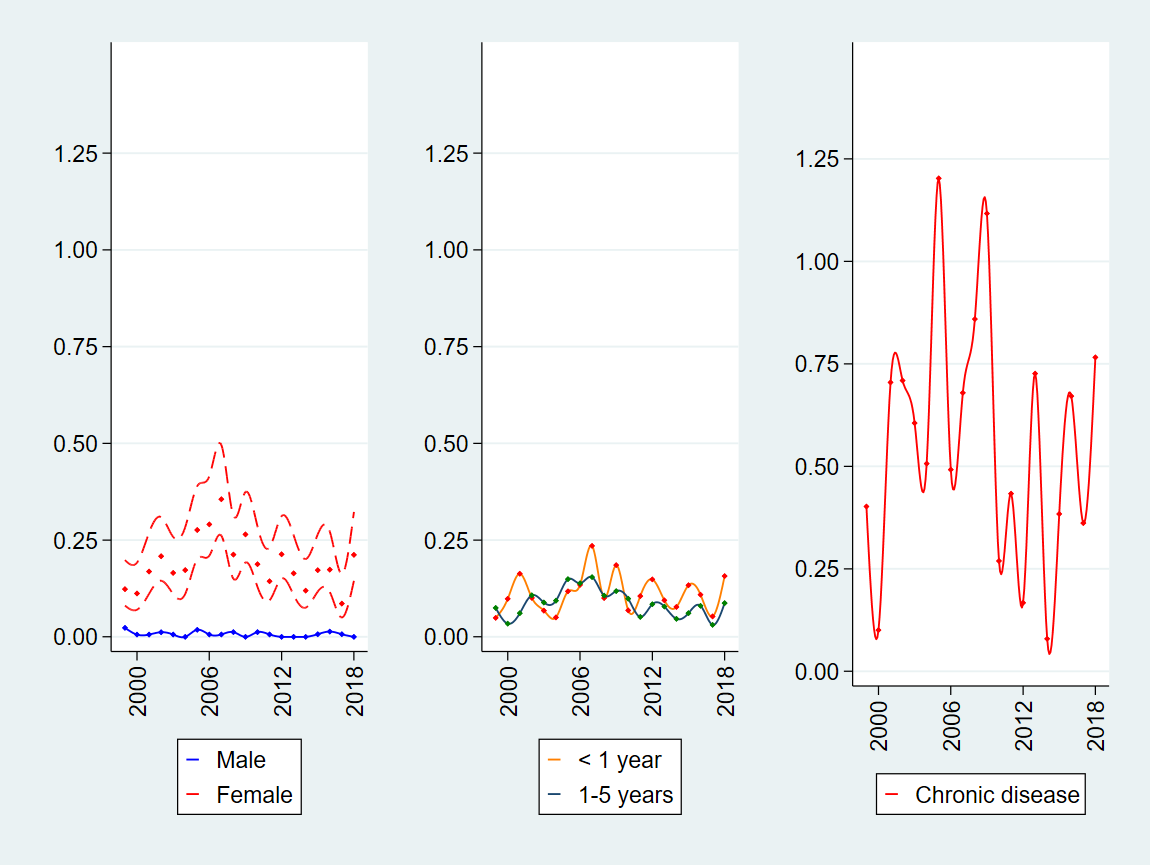


**Fig O in S2 File. Surgical procedures in public hospitals per 1000 person-years, Danish children 0-5 years of age, 1999-2018.** The upper curve represents the overall number of procedures. The lower curves represent the surgical specialties (sorted by incidence in the legend). The 95% pointwise confidence limits and the absolute numbers are only provided for the most frequent procedures to enhance visual appearance.


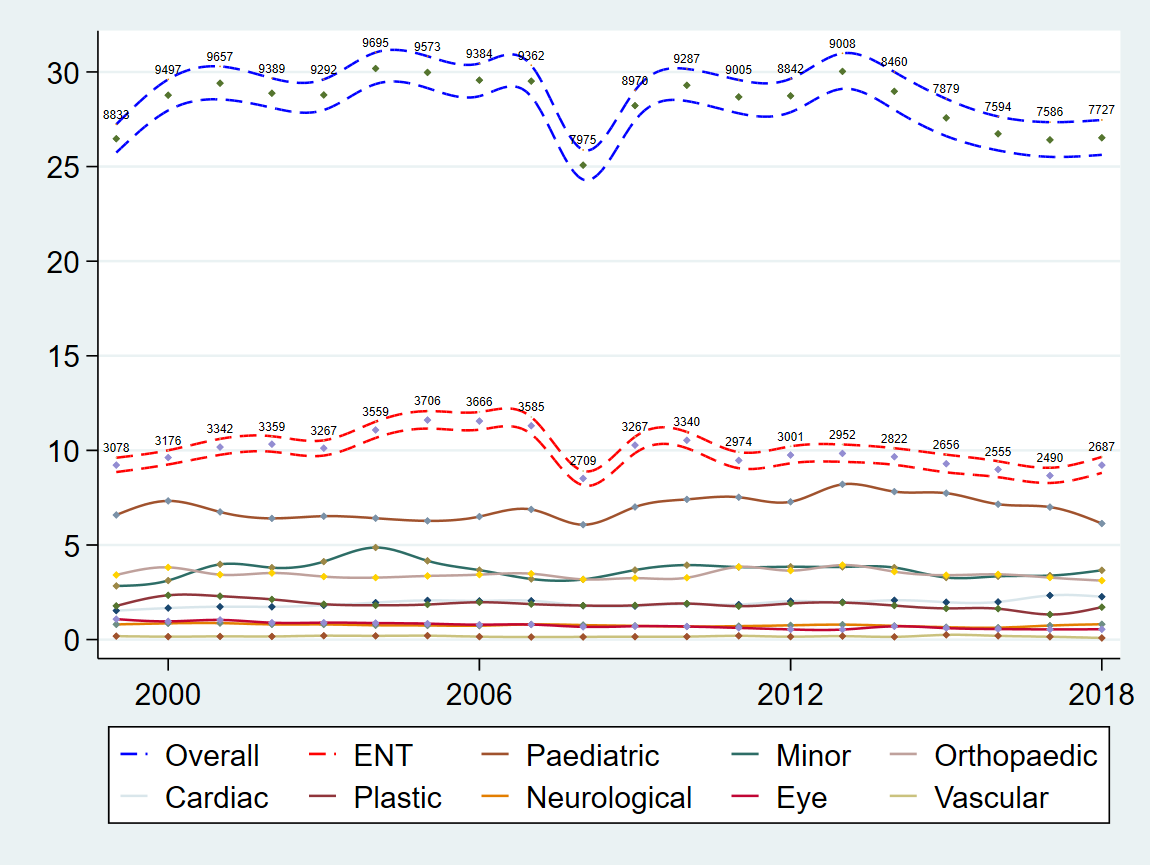

Supplement: S2 File — (DOCX) [file pone.0285047.s002.docx]
