# Supplementary material for: Surgical procedures in Danish children 1999–2018
Source: PLoS One. 2023 Apr 27;18(4):e0285047. doi: 10.1371/journal.pone.0285047 (PMC10138199; doi:10.1371/journal.pone.0285047)
Supplement: S1 Text — (DOCX) [file pone.0285047.s003.docx]

**S1 Text.** **Description of identification of records.**

The objective of this piece of text is to describe in more detail how the information presented in the main paper was gathered. All the data were accessed through Statistics Denmark on a secure server.

The second version of the National Patient Register (LPR2) was used throughout the study period, and thus the table and variable names as well as the data structures were based on LPR2.

**The National Patient Register**

The surgical procedures in public hospitals from The National Patient Register (LPR) were identified as follows. First, the main administrative table LPR_ADM was linked to the satellite table LPR_SKSOPR using the key variable RECNUM which identifies the records. The satellite table SKS_OPR contains information on different procedures including surgical procedures. The surgical procedures were identified as the records for which the first character of the string variable C_OPR attained the value “K”. The variable C_OPR holds information on the NOMESCO-code and the prefix “K” identifies surgical procedures. The date of the surgical procedure was stored in the variable D_ODTO which was used to make sure that the events occurred in the right periods according to both calendar time and age as specified in the study.

Admitted patients (inpatients) were identified by selecting records for which the variable C_PATTYPE attained the value “00”.

The specific surgical specialties were identified using the NOMESCO code values of C_OPR as explained in the manuscript.

Data from the private hospitals were handled analogously the only exception being related to the naming of the datasets, since the private hospital equivalents of LPR_ADM and SKS_OPR were named as PRIV_ADM and PRIV_SKSOPR. As mentioned in the manuscript, these tables were available from 2002 onwards.

**The Health Service Register**

The Danish National Health Service Register contains a variety of data on primary health care. From 1990 to 2005 the table was named as SYSI in Statistics Denmark, whereas from 2005 onwards the table was renamed to SSSY (and the content of the register was modified). Thus, there was an overlap in 2005. In our study the first version, SYSI, was chosen for that specific calendar year. Unfortunately, only the week numbers and not the exact dates of the records in the Statistics Denmark version of the Health Service Register are provided. As a convention the date of the records were set to the Wednesday of the week in question. Records of general anaesthesia were identified by selecting observations for which the variable SPECIALE contained the string “01” as the first two characters and duplicates within individual and date were dropped.

**Death and migration**

The individuals were censored at death or migration. Dates of death were extracted from the table DOD using the variable DODDATO. Dates of migrations were identified using the variable HAEND_DATO from the table VNDS. More specifically, emigrations were identified by selecting records for which the variable INDUD_KODE attained the value “U”.
