# Supplementary material for: Surgical procedures in Danish children 1999–2018
Source: PLoS One. 2023 Apr 27;18(4):e0285047. doi: 10.1371/journal.pone.0285047 (PMC10138199; doi:10.1371/journal.pone.0285047)
Supplement: Attachment — Submitted filename: Response to Reviewers.docx [file pone.0285047.s005.docx]

**General Comment from the Authors**

We thank the editor for the consideration of our revised manuscript. The editor noted that our reply to one of the reviewer points (Q4) was not fully implemented in the revised version. This has been corrected in the re-submitted version as explained below.

**Review Comments to the Author and Author response**

4. It remains unclear why the study period was chosen and why procedures in private hospitals were included only with their availability in the database. Wouldn't it be the more natural choice to start the investigation in 2002 then, because lines 68-70 do not provide a strong justification for the choice of the investigated years.

***Author response: The study period was chosen as 1999-2018 due to data availability and to ensure that all calendar years throughout the study period included children 0-5 years of age. Cf. line 70-73. Apart from that, however, we used the maximal study period to exploit as much information as possible.***

***We acknowledge that the registration of procedures in private hospitals is unclear. We have tried to specify in the revised version (lines 93-95).***

***Manuscript changes:*** *The cohort was based on all Danish children liveborn in the period 1994-2018. Children were followed from birth until five years of age, 31 December 2018, migration, or death, whichever came first. The observation period was chosen as 1999 through 2018 to ensure similar age distributions across calendar years.* *Before 1999 only children less than 5 years of age were present in the cohort. (…)* *However, note that information on private hospitals was only included in The National Patient Register from 2002 onwards and due to initial lack of registrations in the subsequent years, the present study covered the period 2004-2018 for the outcome surgical procedures in private hospitals.*
